# Controlled feature selection and compressive big data analytics: Applications to biomedical and health studies

**DOI:** 10.1371/journal.pone.0202674

**Published:** 2018-08-30

**Authors:** Simeone Marino, Jiachen Xu, Yi Zhao, Nina Zhou, Yiwang Zhou, Ivo D. Dinov

**Affiliations:** 1 Statistics Online Computational Resource, Department of Health Behavior and Biological Sciences, University of Michigan, Ann Arbor, Michigan, United States of America; 2 Department of Microbiology and Immunology, University of Michigan, Ann Arbor, Michigan, United States of America; 3 Department of Computational Medicine and Bioinformatics, University of Michigan, Ann Arbor, Michigan, United States of America; 4 Michigan Institute for Data Science, University of Michigan, Ann Arbor, Michigan, United States of America; University of Ulm, GERMANY

## Abstract

The theoretical foundations of Big Data Science are not fully developed, yet. This study proposes a new scalable framework for Big Data representation, high-throughput analytics (variable selection and noise reduction), and model-free inference. Specifically, we explore the core principles of distribution-free and model-agnostic methods for scientific inference based on Big Data sets. Compressive Big Data analytics (CBDA) iteratively generates random (sub)samples from a big and complex dataset. This subsampling with replacement is conducted on the feature and case levels and results in samples that are not necessarily consistent or congruent across iterations. The approach relies on an ensemble predictor where established model-based or model-free inference techniques are iteratively applied to preprocessed and harmonized samples. Repeating the subsampling and prediction steps many times, yields derived likelihoods, probabilities, or parameter estimates, which can be used to assess the algorithm reliability and accuracy of findings via bootstrapping methods, or to extract important features via controlled variable selection. CBDA provides a scalable algorithm for addressing some of the challenges associated with handling complex, incongruent, incomplete and multi-source data and analytics challenges. Albeit not fully developed yet, a CBDA mathematical framework will enable the study of the ergodic properties and the asymptotics of the specific statistical inference approaches via CBDA. We implemented the high-throughput CBDA method using pure R as well as via the graphical pipeline environment. To validate the technique, we used several simulated datasets as well as a real neuroimaging-genetics of Alzheimer’s disease case-study. The CBDA approach may be customized to provide generic representation of complex multimodal datasets and to provide stable scientific inference for large, incomplete, and multisource datasets.

## Introduction

Data science is an emerging transdisciplinary field connecting the theoretical, computational, experimental, biomedical, social, environmental and economic areas [1]. It deals with enormous amounts of complex, incongruent, and dynamic data from multiple sources and aims to develop algorithms, methods, tools, and services capable of ingesting such datasets and generating semi-automated decision support systems. Predictive analytics is the process of utilizing advanced mathematical concepts, powerful statistical computing algorithms, efficient software tools and services to represent, interrogate, and interpret complex data [2]. As its name suggests, a core aim of predictive analytics is to forecast trends, predict patterns in the data, or prognosticate the process behavior within the range or outside the range of the observed data (e.g., in the future, or at locations where data may not be available) [3]. The increase of the volume and complexity of data outpaces both the growth of computational power needed to extract actionable information from the data as well as the methodological advances needed to interpret the intrinsic characteristics of the observed information.

The proposed Compressive Big Data analytics (CBDA) provides a general foundation for effective representation, efficient processing, and model-free inference for complex heterogeneous data archives. Specifically, CBDA allows us to eliminate noise, forecast trends, compute probabilities, estimate likelihoods, and classify large, incomplete, and heterogeneous data from multiple sources. We demonstrate the utility of CBDA to identify critical data features associated with specific traits, track multivariate relations and predict high-order trends in the data. Complex simulated and observed biomedical data are used to validate CBDA performance.

In this study, big biomedical data is defined as data that exhibits most of the following six characteristics (the six dimensions of Big Data): large size, format heterogeneity and complexity, representation incongruence, incompleteness, multi-scale composition, and multi-source origins [4]. This constructive definition is derived by examining the common characteristics of many dozens of biomedical and healthcare case-studies, involving complex datasets that required special handling, advanced processing, contemporary analytics, interactive visualization tools, and translational interpretation. The definition also identifies methodological gaps, computational barriers and analytical challenges associated with interrogating big biomedical data. Specifically, these challenges include i) infrastructure for transferring, handling, aggregating, processing, and interpreting vast amounts of time-varying data, ii) mathematical foundation for representing and modeling the observed incomplete information, iii) efficient, reliable and precise computational algorithms for statistical analysis, and iv) novel techniques for semi-supervised scientific inference.

There are major challenges and gaps in Big Healthcare Data analytics, including (a) choosing reliable predictive model(s) to apply to the data (e.g., need to define a performance metric), (b) specification and implementation of optimal algorithm(s), (c) feasibility, scalability and convergence of the protocol on large datasets, and (d) access to appropriate computational resources. The compressive big data analytic (CBDA) technique tackles most of these challenges.

In this manuscript, we attempt to address some of the above stated challenges by developing an end-to-end computational protocol that includes data ingestion, harmonization, preprocessing, analysis, inference and interpretation. Two open-source implementations of the CBDA protocol are available—platform-agnostic stand-alone R package (https://cran.r-project.org/package=CBDA) as well as a reproducible pipeline graphical workflow (wrapper of the R-package). Following FAIR (Findable, Accessible, Interoperable, Reusable) principles for data sharing and in accordance with open-science community standards [5], all of our work is freely available for independent validation, results reproducibility, independent extension and validation (https://github.com/SOCR/CBDA).

To validate CBDA, we compare it to Knockoff filtering [6, 7], which is a novel controlled variable selection statistical technique using FDR (False Discovery Rate). Knockoff filtering doubles the number of original features by introducing null-features (${\tilde{x}}_{j}$) corresponding to all features (*x*_*j*_) in the original design matrix X. The extra decoy (knockoff) variables serve as a "control group" that allows estimation of the rate at which the regularized linear modeling generates false-positive variable-selection results. S2 Text provides additional knockoff practical and mathematical details. Briefly, knockoff filtering has advantages such as computational efficiency—decoy feature construction does not require any new data, and flexibility—the technique allows specification of a range of test statistics. However, it requires the number of the features to be smaller than the number of the cases. A newer model-free knockoff version was recently described [8], however, the R package implementation has not yet been released.

Similar to CBDA, Bagging and RandomForest techniques [9, 10] also use the core principle of *subsampling* to improve the model prediction. However, there are CBDA differences in the goals and the specific model averaging methods used following the stochastic sample generation. For instance, bagging averaging typically involves ${\hat{f}}_{bag}\left(x\right)=\frac{1}{B}\Sigma_{b=1}^{B}{\hat{f}}^{*b}\left(x\right)$ prior to obtaining the final prediction model, whereas CBDA subsampling targets feature selection. For low signal-to-noise ratio (noisy data), RandomForest also may introduce overfitting, increasing the salience of selected wide-range features.

The main differences between CBDA and other subsampling techniques are twofold. First, CBDA relies on a Divide-and-Conquer strategy to iteratively obtain and organize the representative data samples. This facilitates stochastic sample-based data-driven inference, just like compressive sensing does for signal reconstruction. Second, CBDA does not make assumptions about the data homologies, feature consistency or completeness. It represents a model-free technique that iteratively harmonizes and ensembles data to provide sample-driven inference or parameter estimation.

**Fig 1:**
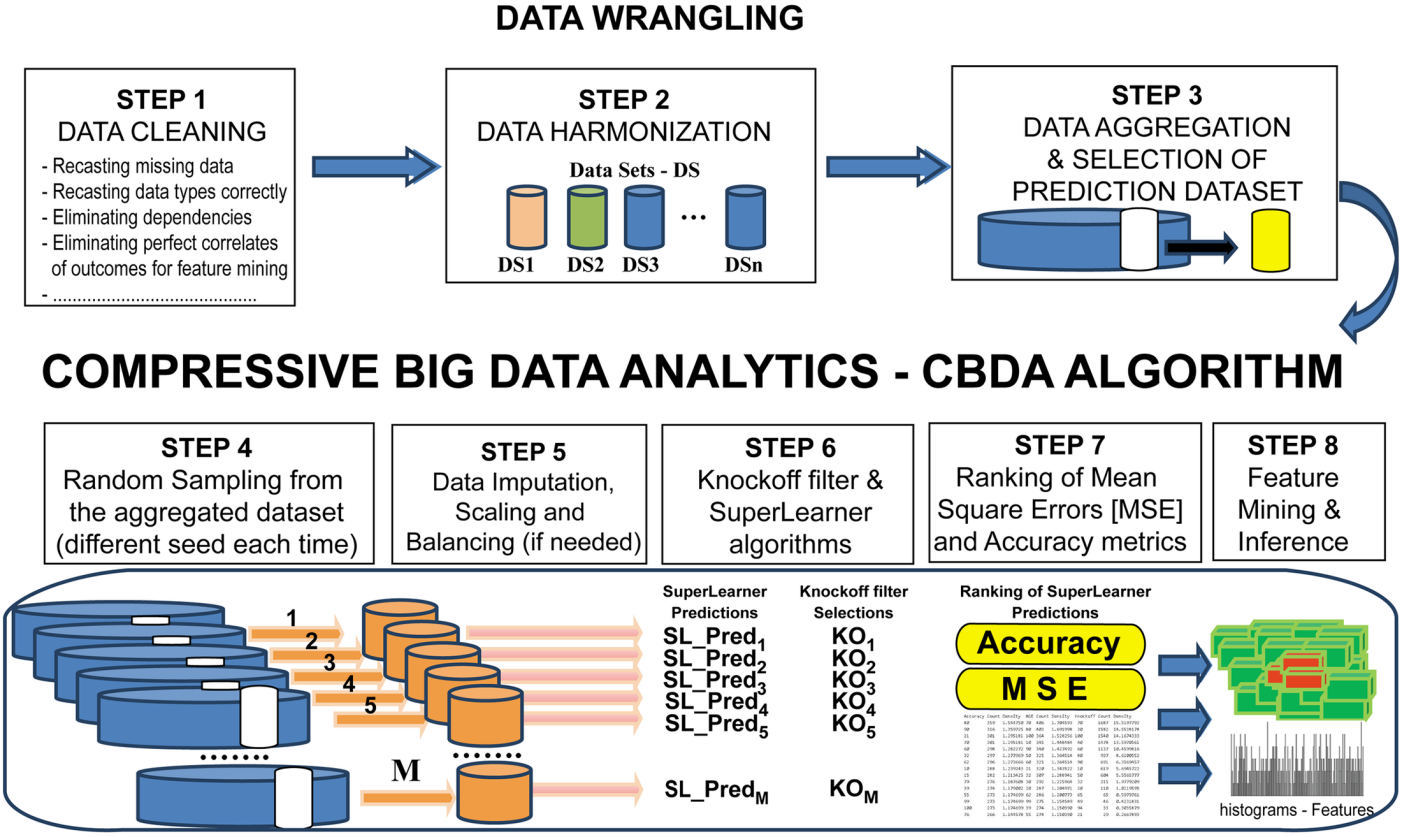
CBDA framework. CBDA involves the following steps: *Step1*: Data Cleaning, *Step 2*: Data Harmonization, *Step 3*: Data Aggregation and Selection of Prediction Dataset. The first three steps represent Data Wrangling. *Step 4*: Random Sampling from the aggregated dataset, *Step 5*: Data Imputation, Scaling and Balancing (if needed), *Step 6*: Controlled variable selection and SuperLearner algorithms, *Step 7*: Ranking of Mean Square Errors (MSE) and Accuracy metrics, and finally, *Step 8*: Feature Mining and Inference.

We first present the foundation of compressive big data analytics (CBDA). Then, we describe one specific CBDA implementation, apply the technique to simulated and real data, and compare CBDA against some alternative methods. The CBDA protocol is illustrated in Fig 1. Its feasibility and scalability are ensured by the *Divide-and-Conquer strategy* (see Methods section for details), where a very large training set is reduced to smaller chunks by iteratively sampling with replacement features and cases following certain input specifications. These smaller training sets are then analyzed by an ensemble predictor that combines many different pre-defined algorithms into a single predictive model (i.e., the SuperLearner-SL, see [11, 12] for details). By using the SL algorithm, we greatly simplify the choice of the predictive model(s) to apply to the data. We accomplish that by using the large selection provided by the SuperLearner library (see Methods section and [11–21] for details on the many different algorithms used in our CBDA protocol). The default algorithms specifications can be easily expanded from the default values, bypassing the uncertainty associated to the selection of the most appropriate algorithm for the data under analysis. The best algorithm(s) within the ensembles can always be retrieved as an output, thus suggesting future direction for parameter identification/estimation of mechanistic models. To ensure the method reliability, we employ False Discovery Rate (FDR) controlled variable selection, which may be implemented using the knockoff (KO) filter algorithm, see [6] for details. By combining R tools (e.g., SuperLearner) [11] with the LONI pipeline environment for distributed computing [22] we develop a novel protocol for (a) effective and reproducible analysis of diverse datasets, (b) comparing selected key features/biomarkers across different methods or experiments, and (c) enable convergence tracking, large-scale testing and validation.

## Methods

This section illustrates the CBDA methodology for representing and analyzing large datasets with binomial/multinomial outcomes. First, we describe the protocol steps and then outline the validation procedure using synthetic and clinical datasets. In support of transparent, reproducible, and open-science principles, the CBDA protocol has been developed in the R environment (https://www.r-project.org). Since a large number of smaller training sets are needed for the convergence of the protocol, we created a workflow that runs on the LONI pipeline environment (http://pipeline.loni.usc.edu), a free platform for high performance computing that allows the simultaneous submission of hundreds of independent instances/jobs of the CBDA protocol (see [22] for details). The methods, software and protocols developed here are openly shared on our GitHub repository (https://github.com/SOCR/CBDA). All software, workflows, and datasets are publicly accessible. The CBDA protocol steps are illustrated in Fig 1. Detailed descriptions of the protocol are given in the next sections and in S1 and S2 Text.

### CBDA data wrangling

The CBDA protocol starts with three modules that are data dependent, namely Data Cleaning, Harmonization, and Aggregation (see Fig 1). These first three modules represent standard procedures and techniques in data wrangling (see [23] for details). Different datasets will require ad hoc data wrangling procedures and cannot be all comprehensively generalized. Each dataset is labeled as *DSorigin*_*p*_ (*p* = 1,2, …, *n*). We will use the terms rows/cases and columns/features to indicate the dimensions of each dataset. **Step 1 Data cleaning** ensures that: (i) all the data types are correctly interpreted, e.g., casted into the R data frame; (ii) missing values are correctly identified and labeled; (iii) known dependencies are addressed, e.g., eliminating the features that are perfectly correlated to each other and/or to the outcome/response variable; and (iv) identifying and eliminating static features, e.g., constant values.

**Step 2 Data Harmonization** is performed by identifying features that are common across different datasets, ensuring they are consistently casted across datasets (i.e., double, integer, categorical). **Step 3 Data Aggregation and Selection of Prediction Dataset** follows on harmonization, by merging multiple datasets together correctly (for example, using common features as keys). At the end of Step 3, a prediction set is randomly sampled from the dataset and held off for validation (i.e., [*X*_*val*_, *Y*_*val*_], usually 20% of the original number of rows/cases). We set the random seed to a fixed value to enforce reproducibility of the results, and at the same time, to comply with the requirement that no validation data can be used for training.

### CBDA sampling scheme

Following **Steps 1–3**, we have set aside the prediction dataset for validation and we are left with a subset of the original Data labeled training set [*X*_*temp*_, *Y*_*temp*_]. The next steps of the CBDA protocol are data independent and pertain training/learning and feature mining. They can be applied to any type of dataset, as long as the data wrangling steps have been performed successfully.

During the learner training process, **Step 4 Random Sampling** ensures that the large training set [*X*_*temp*_, *Y*_*temp*_] is reduced to smaller chunks by defining ranges from which to select a certain fraction of cases (Case Sampling Range—CSR) and features (Feature Sampling Range—FSR). Each sampling step is with replacement and it returns *M* subsets of cases/rows *n*_*j*_ and features/columns *k*_*j*_ that are used to build the smaller training sets [*X*^*j*^, *Y*^*j*^]_*j* = 1,2,…, *M*_. If needed, **Step 5 Data Imputation** (see [24] for details), **Scaling** (see [25] for details) and **Balancing** (see [26] for details) are performed on the chunk training set [*X*^*j*^, *Y*^*j*^] (see S2 Text) for details on the algorithms used). More details are given in the CBDA Testing Protocol section.

### CBDA predictive components

**Step 6 Knockoff filter and SuperLearner algorithms**. The training set pair [*X*^*j*^, *Y*^*j*^] is then passed to the SuperLearner (see [11] and S2 Text for details) and to the knockoff filter (see [6] and S2 Text for details) algorithms, respectively. The SuperLearner is an ensemble predictor that combines many different algorithms into a single predictive model (see S2 Text for details on the algorithms used). The SuperLearner function takes the training set pair [*X*^*j*^, *Y*^*j*^] and returns the predicted values based on the validation data *X*_*val*_. The knockoff filter algorithm takes the training set pair [*X*^*j*^, *Y*^*j*^] and returns a subset of features from *X*^*j*^ selected as most important in explaining *Y*^*j*^.

**Step 7 Ranking of MSE and Accuracy metrics**. While the results of the Knockoff filter already return a set of features as likely most important (e.g., here we use the Knockoff filter as a benchmark to test the CBDA accuracy), the SuperLearner function returns predictions that we rank based on two metrics: accuracy of predictions (highest to lowest) and mean square errors (MSE) from the predictions (lowest to highest). The accuracy metric is generated by calculating a confusion matrix on the *Y*_*val*_ and the $\hat{Y^{j}}$ (see **mathematical framework** for details) and retrieving the accuracy of the prediction (see S2 Text for details on the confusionMatrix function). The MSE metric is generated by calculating the Euclidean distance between the *Y*_*val*_ binary values and the probability predictions returned by the SuperLearner. We perform feature mining on the SuperLearner predictions by first extracting the features *f*_*j*_ from the top-ranked predictions (since each prediction is associated with a subset of features selected in Step 4 of the CBDA). Then we choose how many top-ranked predictions to consider and calculate the frequencies each feature occurs among these top-ranked predictions. In **Step 8 Feature Mining**, again, we rank the top predictions based either on the highest accuracy or on the lowest MSE. Then, we calculate the densities of the features among the top-ranked. If a feature or set of features is associated to the top-ranked predictions, we will see spikes in the correspondent generated histograms.

Below, we show a concise description of CBDA framework:

**Step1-Step5** are described in the **mathematical framework (Supplementary Materials)**.

**Step 6**: **Algorithm**

- Start with a generic dataset: [*X*, *Y*, *X*_*val*_, *Y*_*val*_], let $C^{j}\;=\;[X^{j},Y^{j},X_{val}^{j}]$, *j* = 1,…, *M*.
- Define machine learning algorithm as ML: $ML(C^{j}):R^{n_{j}\times k_{j}}\times R^{n_{j}}\times R^{m\times k_{j}}\to R^{m}$, $ML(\left[X^{j},Y^{j},X_{val}^{j}\right])\;=\;\hat{Y^{j}}.$

**Step 7**:

- Define performance metric as: *τ*(*ML*(*C*^*j*^), *Y*_*val*_): *R*^*m*^ × *R*^*m*^ → *R*, $\tau (\hat{Y^{j}},Y_{val})\;=\;c_{j},\;$ *j* = 1,2, …, *M*.
- Rank the samples: {*c*_(*j*)_} = *O*^*q*^ ({*c*_*j*_}), *j* = 1,2, …, *q*.

**Step 8**:

Set the feature values: $s_{j}^{\left(i\right)}=Dirichlet_{j}\left(f_{i}\right)=\{\begin{array}{l} 0,\;f_{i}\notin sample\;j\\ 1,\;f_{i}\in sample\;j \end{array}.$

- Set a metric as: *F* ∈ *R*^*q*×*K*^, $\forall b_{ji}\in F,b_{ji}\;=\;s_{j}^{\left(i\right)}$.
- Count the occurrence: $S_{i}\;=\;\sum_{j}^{q}b_{ji},i\;=\;1,2,\;.\;.\;.,K$.
- Feature mining: *S*_(*i*)_ = *O*^*K*^ (*S*_*i*_), *i* = 1,2, …, *K*, Ω* = {*f*_1_, …, *f*_*i*_, …, *f*_*K*_}.
- Inference: let *C*^*p*^ = [*ϕ*_*p*_ *X*, *ϕ*_*p*_ *Y*, *ϕ*_*p*_ *X*_*val*_] and $[ML_{p}\left(C^{p}\right):R^{n\times k_{p^{*}}}\times R^{n\times 1}\times R^{m\times k_{p^{*}}}\to R^{m\times 1}\to R^{m\times 1}$, $ML\left(\left[\Phi_{p}X,\Phi_{p}Y,\Phi_{p}X_{val}\right]\right)=Y_{val}^{p},p=5,10,\ldots ,\hat{p}$, $\tau \left(Y_{val}^{p},Y_{val}\right):R^{m}\times R^{m}\to R$, $\tau \left(ML\left(\left(\Phi_{p^{*}}X,\Phi_{p^{*}}Y,\Phi_{p^{*}}X_{val}\right)\left[\Phi_{p^{*}}X,\Phi_{p^{*}}Y,\Phi_{p^{*}}X_{val}\right]\right),Y_{val}\right)=\underset{p}{\text{best}}\left(\tau \left(ML\left(\Phi_{p}X,\Phi_{p}Y,\Phi_{p}X_{val}\right),Y_{val}\right)\right)$.
- Then *Φ*_p*_ is the final dictionary we need.
- At the end, we assess the CBDA performance. Once we obtain *Φ*_p*_, we can use it along with the SuperLearner algorithm and the testing set predictors *X*_*exp*_ to estimate predicted outcome: *Y*_*exp*_ = *ML*([*Φ*_p*_ *X*, *Φ*_p*_ *Y*, *Φ*_p*_ *X*_*exp*_]).

### Datasets

We validate the CBDA technique on three independent datasets. The first two, namely the Null and Binomial datasets, are synthetically generated as cases (i.e., *n*) and features (i.e., *p*) for the purpose of testing the protocol and assessing the CBDA performance. For all the Binomial datasets, only 10 features are used to generate the outcome variable (these are what we call truly predictive features, see details below in the Binomial Datasets section). The third case-study represents a real biomedical dataset on Alzheimer’ss disease using clinical and neuroimaging measures. These data archives include appropriate and relevant categorical (binomial/binary and multinomial/polytomous) outcome features.

#### Null datasets

The first set of data is a "white noise" dataset (i.e., Null dataset), where the outcome Y is a realization of a Bernoulli vector of length *n* (i.e., *Y* = [*Y*_1_, *Y*_2_, …, *Y*_*n*_], with *Y*_*i*_~*Bernoulli*(0.5), *i* = 1,2, …., *n*) completely independent from the set of features X. Each column of X is an independent realization of a Gaussian random variable with mean equal to 0 and standard deviation equal to 1 (i.e., *X* = [*X*_1_, *X*_2_, …, *X*_*p*_], with *X*_*j*_ ∼ *N*(0,1), *j* = 1,2, …, *p*. We will refer to *n* as number of cases and to *p* as number of features. We use different ratios *n/p* for a more complete assessment of the robustness and convergence of the CBDA protocol in the binomial case. Namely, we use *n/p* ratios of 1/3 (100/300), 3 (900/300) and 5 (1,500/300). We also superimpose an artificial fraction of missing data on these datasets to test the imputation procedure itself. This superposition of missing data is accomplish using missing completely at random (MCAR) sampling, via the R function *'prodNA'*. This process artificially introduces missing values by deleting the data elements at the specified indices, i.e., we introduce MCAR NAs in a given data frame according to the desired missingness fraction.

#### Binomial datasets

The second set of data is similar to the Null dataset, but the Bernoulli vector Y is now an explicit function of the set of features X. We establish the dependency of Y to X by selecting 10 features from X to build a linear additive model *Y* ∼ *X*, with non-zero coefficients for only these 10 features, namely $Z\;=\;b_{k_{1}}X_{k_{1}}+b_{k_{2}}X_{k_{2}}+b_{k_{3}}X_{k_{3}}+\;.\;.\;.+b_{k_{10}}X_{k_{10}}+e,\;with\;e∼N(0,1)\;and\;b\;=\;b_{k_{j}}\;(j\;=\;1,2,\;.\;.\;.,10))$. The Bernoulli outcome Y is then generated by an inverse logit on the outcome of the linear additive model (i.e., $Pr\;=\;\frac{1}{1+e^{-Z}}$ and *Y*_*i*_~*Bernoulli*(*Pr*), *i* = 1,2, …., *n*). When necessary, various strategies may be used to binarize the predicted outcomes using the corresponding probability values. Similar to the Null dataset, we superimpose MCAR missingness on this dataset to test the reliability of the imputation procedure.

#### Alzheimer’s Disease Neuroimaging Initiative (ADNI) case-study

This dataset includes clinical and neuroimaging data for a cohort of elderly volunteers. It consists of three cohorts of patients (2,500 cases) with three diagnoses (i.e., multinomial): EO-AD (Early Onset Alzheimer Disease, N1 = 406), Normal (N2 = 747) and EO-MCI (Early Onset Mild Cognitive Impairment, N3 = 1,347). We used both Global (GSA) and Local (LSA) Shape Analysis neuroimaging biomarkers based on 56 region of interests (ROI) described in the LONI Probabilistic Brain Atlas (LPBA, see [27] for details). The necessary data wrangling (Step 1) was performed on the ADNI data. There were redundant and/or highly-correlated features, among the predictors as well as between predictors and the outcome, i.e., diagnosis. Following Steps 1 and 2 of the CBDA protocol (see Fig 1), the SuperLearner and Knockoff algorithms were employed to predict the participant clinical diagnosis. Table 1 shows a summary of Alzheimer Disease Neuroimaging Initiative (ADNI) archive, while S2 Table shows a list of all the features in the dataset.

**Table 1:** Alzheimer Disease Neuroimaging Initiative dataset.

Source	Types of Data	Sample Size	Clinical Relevance
**ADNI Archive** www.adni-info.org	**Clinical data:** demographics, clinical assessments, cognitive assessments **Imaging data:** sMRI, fMRI, DTI, PiB/FDG PET **Genetics data:** Ilumina SNP genotyping **Chemical biomarker:** lab tests, proteomics	Each data modality comes with a different number of cohorts. Generally, 500–2,500 subjects (for instance see [27–29] for previously conducted ADNI studies	ADNI provides interesting data modalities, multiple cohorts (e.g., early-onset, mild, and severe dementia, controls) that allow effective model training and validation

### CBDA testing protocol

Table 2 summarizes all the specifications used to initialize each experiment, as well as all the options we implemented for the post-optimization analysis, where we rank all the predictions and mine for key features. For the Null and Binomial test datasets, an experiment is defined as an entire set of 9,000 jobs and it is uniquely identified by a set of input specifications that are read from an argument file.

**Table 2:** Input specifications for all CBDA experiments used to validate the convergence of the CBDA method.

M [number of CBDA iterations]	Fraction of missing values [misValperc]	Feature Sampling Range [FSR]	Cases Sampling Range [CSR]	Subsets of M	Top-Ranked Predictions
9,000	0% 20%	[1%,5%] [5%,15%] [15%,30%]	[30%,60%] [60%,80%]	1,000 3,000 6,000 9,000	100 200 500 1,000

The array of input specifications comprises the following labels:

1. M: number of the instances/jobs for each experiment (set to 9,000 in this study)
2. misValperc: % of missing values to introduce in the Data (just for testing, to mimic real cases)
3. min_FSR: Lower bound for the % of features/columns sampling
4. max_FSR: Upper bound for the % of features/columns sampling
5. min_CSR: Lower bound for the % of cases/rows sampling
6. max_CSR: Upper bound for the % of cases/rows sampling

The argument file has as many rows as the number of experiments we want to perform on a single dataset. For each dataset, we run 12 different experiments, combining the fraction of missing values (misValperc), the FSR, and CSR (see Table 2). Thus, each row of the argument file will have the following values [M, misValperc, min_FSR, max_FSR, min_CSR, max_CSR].

Sampling ranges for cases (CSR—Cases Sampling Range) and features (FSR—Feature Sampling Range) are then defined as follow: FSR = [min_FSR, max_FSR] and CSR = [min_CSR, max_CSR] (see Table 2 for the options we investigated).

Depending on the number of features, the lower bound for FSR can be set to include at least 5–10 features. So, for example, if we have only 100 features in the dataset, a lower bound of 1% is not feasible, since it would only select 1 feature for the CBDA protocol.

**Fig 2:**
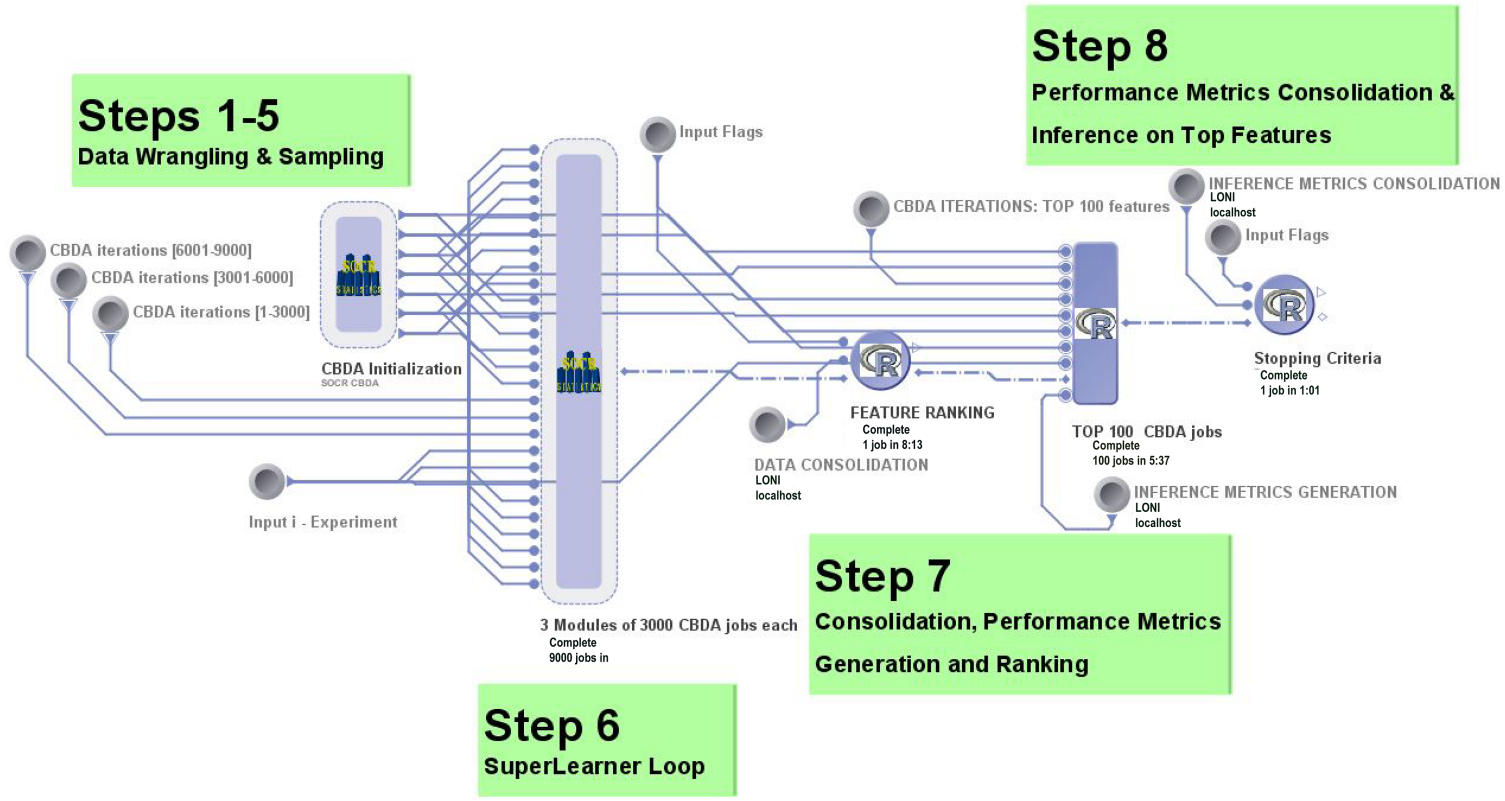
LONI pipeline workflow for the CBDA protocol. In the graphical pipeline workflow implementation, the CBDA technique is divided into following steps. Step 1–5 is data wrangling and sampling; Step 6 represents the SuperLearner loop; Step 7 is consolidation, performance metrics generation, and ranking; and Step 8 includes consolidation of performance metrics and inference on the top features.

For the ADNI dataset, we did not introduce artificial missing values (i.e., misValperc = 0%), and we did not implement the FSR ranges [1%-5%] and [5%-15%] because the data wrangling steps reduced the viable features for learning down to 68. Thus, for the ADNI dataset we only performed 4 experiments, only varying CSR = [min_CSR, max_CSR] and FSR = [15%, 30%]. To investigate the convergence of the CBDA within the context of the Binomial datasets, we chose to select subsets of M for the ranking, namely the first 1,000, 3,000, 6,000 and then all 9,000 samples. The specifications for ranking the top predictions are given in the last column of Table 2. We selected 100, 200, 500 or 1,000 top-ranked predictions. Fig 2 showcases all the possible combinations of the latter two specifications (for a total of 16 combinations, 4x4) for each experiment in each Binomial dataset. Table 2 summarizes the experimental design specifications for the CBDA protocol, and should be used as a guideline to navigate through all the Results section. Our goal was to investigate the optimal input specifications for the CBDA protocol (i.e., decrease the computational time by reducing the number of samples, and increase the rate of discovery of "true" features).

### High performance computing

The feasibility of the CBDA protocol is significantly enhanced by utilizing a high-throughput, scalable, efficient and fast computational infrastructure to manage the tens of thousands of processing tasks that collectively represent the entire CBDA method. We have chosen to implement CBDA as a graphical workflow that can submit thousands of [*X*_*j*_, *Y*_*j*_], analysis/jobs simultaneously via the LONI pipeline environment (http://pipeline.loni.usc.edu and [22]). The R script that implements the CBDA protocol is generalized so that the job identifier (i.e., label j) is passed to the script together with other inputs (see the pseudocode in S1 Text for details) to build a single instance of the CBDA protocol. Once the SuperLearner prediction and the knockoff filter are generated, a workspace is saved and the instance is completed. A post-optimization workflow is executed once all the instances M are completed: it consolidates and ranks all the metrics into a single R workspace. An offline R markdown script then generates histograms and table with results.

Due to some restrictions on the LONI cranium server, our submission queue is limited to 3,000 instances at a time. To investigate asymptotic convergence properties of the CBDA protocol, we decided to set the total number of instances (i.e., jobs) for each CBDA analysis to 9,000. Fig 2 shows an example of the workflow as implemented in the LONI pipeline GUI client.

Each job completes within 5–10 minutes upon submission, which makes the CBDA protocol very scalable and efficient. Table 3 shows some computational complexity estimates of the CBDA protocol in three different scenarios: desktop/laptop, small and large multicore servers.

**Table 3:** CBDA computational complexity.

CBDA Computational Complexity	CPU time per job	Total CPU time (M = 9000)
Desktop/Laptop	~3–10 mins	[x M] ~450–1500 hrs
Small Multicore Server (# cores n ~20–30)	~3–10 mins	[(x M)/n] ~15–75 hrs
Large Cloud Server (Cranium, # cores n ~3000 cores)	~3–10 mins	[(x M)/n] ~15–20 mins

## Results

Our experimental design aims to empirically investigate the convergence properties of the CBDA in mining true predictive features, and more specifically, determine which metric is best suited to assess CBDA performance. We applied our CBDA protocol to two simulated datasets, *Null* and *Binomial*, where we control the model that generated the data as well as the number of true predictive features. The simulated Binomial datasets represent a true positive validation example. Then, we will contrast these results to the Null datasets results and estimate the empirical false discovery rate (or true negative rate) for null-feature selection (i.e., the false selection of features in the featureless null dataset). The Null data also allows us to examine the CBDA computational complexity and its robustness in a pure noise scenario. The third dataset represents a real biomedical case-study using data from the Alzheimer’s Disease Neuroimaging Initiative (ADNI) [30]. The details about each dataset are provided in the Datasets section of the Methods. Throughout this section, we will use the knockoff filter [6] as a benchmark for false discovery rate based controlled feature selection.

### CBDA results using binomial data

**Fig 3:**
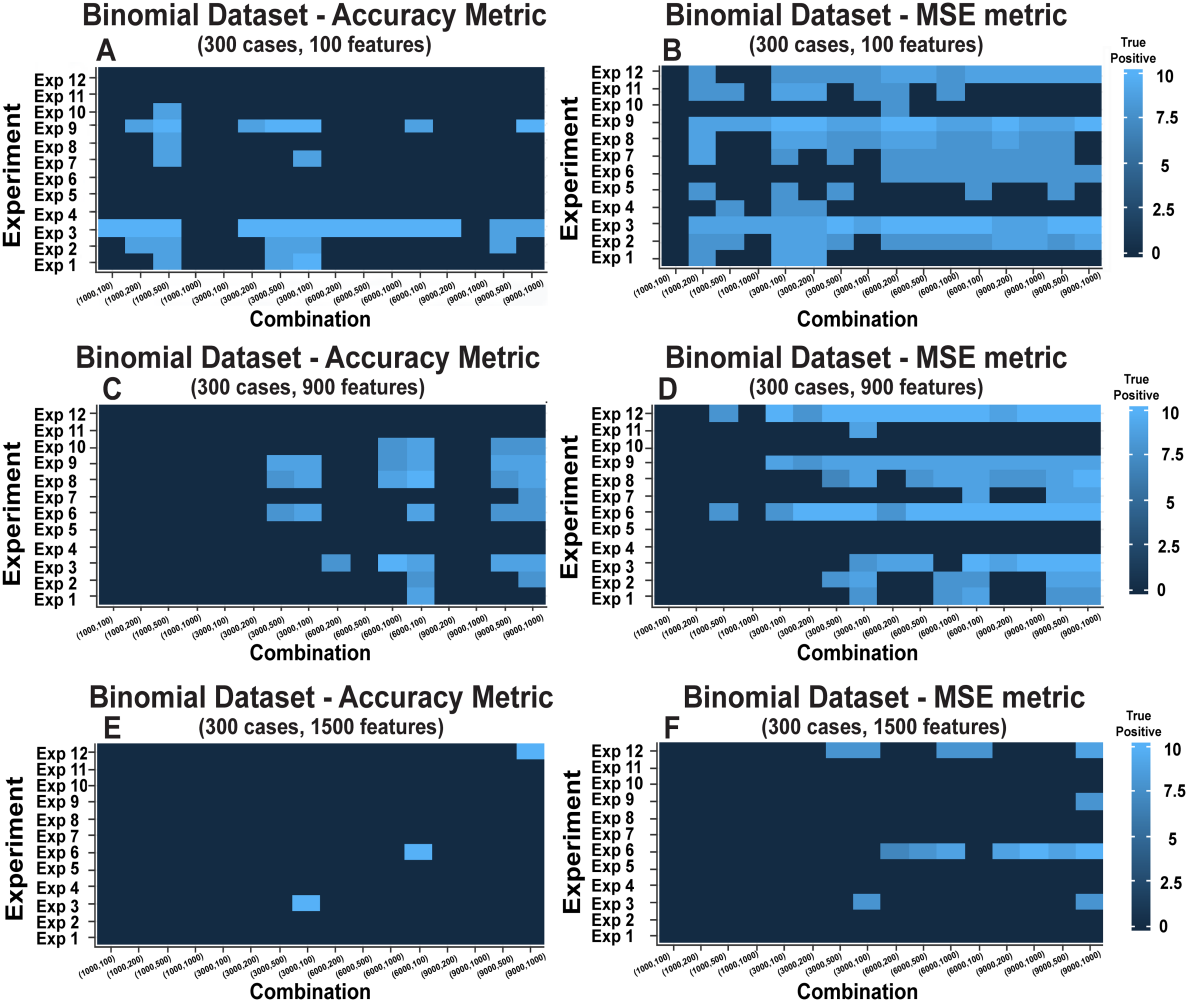
Heatmaps of CDBA protocol for the binomial datasets. The x axis represents the 16 combinations between the choice of the subsets of M (i.e., 1,000, 3,000, 6,000 and 9,000) and the choice for top-ranked predictions (i.e., 100, 200, 500 and 1,000, as described in the last 2 columns of Table 2 in the Methods section). Namely, the combinations are ordered as follows: Combination 1 = (1,000,100), Combination 2 = (1,000,200), Combination 3 = (1,000,500), Combination 4 = (1,000,1,000), Combination 5 = (3,000,100), Combination 6 = (3,000,200), Combination 7 = (3,000,500), Combination 8 = (3,000,1,000), Combination 9 = (6,000,100), Combination 10 = (6,000,200),Combination 11 = (6,000,500), Combination 12 = (6,000,1,000), Combination 13 = (9,000,100), Combination 14 = (9,000,200), Combination 15 = (9,000,500), Combination 16 = (9,000,1,000). The y axis represents the CBDA experiment specs, where Experiments 1–6 have no missing values (i.e., *missValperc* = 0%), and Experiments 7–12 have 20% missing values (i.e., *missValperc* = 20%). Both sets of experiments have the FSR and CSR ranges combined in ascending order, namely Exp1and Exp 7 = [FSR,CSR] = [1–5%,30–60%], Exp2 and Exp 8 = [FSR,CSR] = [5–15%,30–60%], Exp3 and Exp 9 = [FSR,CSR] = [15–30%,30–60%], Exp4 and Exp 10 = [FSR,CSR] = [1–5%,60–80%], Exp5 and Exp 11 = [FSR,CSR] = [5–15%,60–80%], Exp6 and Exp 12 = [FSR,CSR] = [15–30%,60–80%]. See Table 2 for details. Panels A, C and E show the CBDA results using the Accuracy performance metric. Panels B, D and F show the CBDA results using the Mean Square Error-MSE performance metric (see Methods for details on the performance metrics). Panels A and B, C and D, E and F show the results for the 3 Binomial datasets tested, respectively.

Due to the large number of experiments and the many different specifications, the complete set of results for the three binomial datasets are illustrated in S3 Text. Fig 3 includes a summary of all these experiments, namely the analysis of three binomial datasets, 12 experiments and 9,000 CBDA samples for each experiment. At the top of each panel in Fig 3 we show the number of cases (i.e., *n*) and features (i.e., *p*) in the dataset. This figure summarizes in light blue color CBDA experiments with high true positive rates, indicating a high frequency of correct feature identification (i.e., with more than 7 true features identified out of a total of 10) and in dark blue color CBDA experiments with low true positive rates, depicting a low frequency of correct feature identification (i.e., with less than 7 true features identified out of a total of 10). Details of all the experiment specifications are provided in the Methods section.

Briefly, a single CBDA experiment is performed following certain inputs, such as the total number of samples performed (i.e., *M*), the fraction of artificial missingness introduced (*missValperc*), the sampling rates for cases (*Case Sampling Range–CSR*) and features (*Feature Sampling Range—FSR*). The complete set of results used to generate each experimental combination shown in Fig 3 is available on our GitHub repository (https://github.com/SOCR/CBDA). To generate Fig 3, a total of 324,000 CBDA instances were staged and completed on the USC Cranium distributed Pipeline server, Cranium [22]. For each heatmap in Fig 3, the *x* axis represents all the 16 combinations between the choice of the subsets of *M* (i.e., 1,000, 3,000, 6,000 and 9,000) and the choice for top-ranked predictions (i.e., 100, 200, 500 and 1,000, as described in detail in the last 2 columns of Table 2, see Methods section). The combination label lists the pairs [subsets of M, Top-ranked predictions]. For example, Combination 1 has the lowest values for the combined pair (i.e., [1,000,100]), while Combination 16 has the highest values (i.e., [9,000, 1,000]), see legend of Fig 3 details. The maximum count for each cell in the heatmaps is 10 (light blue color), corresponding to identifying all “true” 10 features used to generate the binomial outcome among the top 15 features selected by CBDA for each experiment and each combination. Our minimal target level of true positives in each experiment is 7, thus anything below 7 is marked as 0 in the heatmap (shown as a dark blue spot). An optimal recipe would return the most "true" features selected with the minimum computational complexity (i.e., lowest subsets of M ~3,000). Since our study is focused more on compressing the sampling over the features, rather than over the cases, an assessment on the efficiency of the CBDA protocol is based on spots in the heatmaps with the lowest FSR ranges (i.e., Experiments 1, 4, 7 and 10, where FSR = [1%-5%]).

The first finding of this large experiment is that the mean square error (MSE) metric is more effective in increasing the CBDA performance. This corresponds to observing more light blue spots in the MSE heatmaps compared to the corresponding heatmaps based on the Accuracy measure. The second observation is that with an increased number of features (from 100 to 900, up to 1,500), the CBDA has less optimal combinations that reach our minimal target level of true positives. In this experiment, we did not change the number of "true" features across datasets (there were always 10 true features present in the data). The third finding is that across the 3 binomial datasets, certain experiments (i.e., FSR and CSR pairs) are performing consistently better, e.g., Experiments 3, 6, 9 and 12 (where we used the highest FSR~[15%-30%] and CSR~[60%-80%] combinations). However, for features between 100 and 900, experiments with the lowest FSR (FSR~[1%-5%], e.g., Experiments 1, 4, 7 and 10), are often selected as optimal. Fig 3 suggests that we should choose our CBDA protocol specifications based on the number of features in our dataset and on an a priori assumption that a maximum of 10 features are to be selected for our predictive model. Further analyses and tests will be performed in order to generalize our conclusions based on a signal/noise ratio between true features and total features in the dataset.

### Comparison of the null and binomial data results

**Fig 4:**
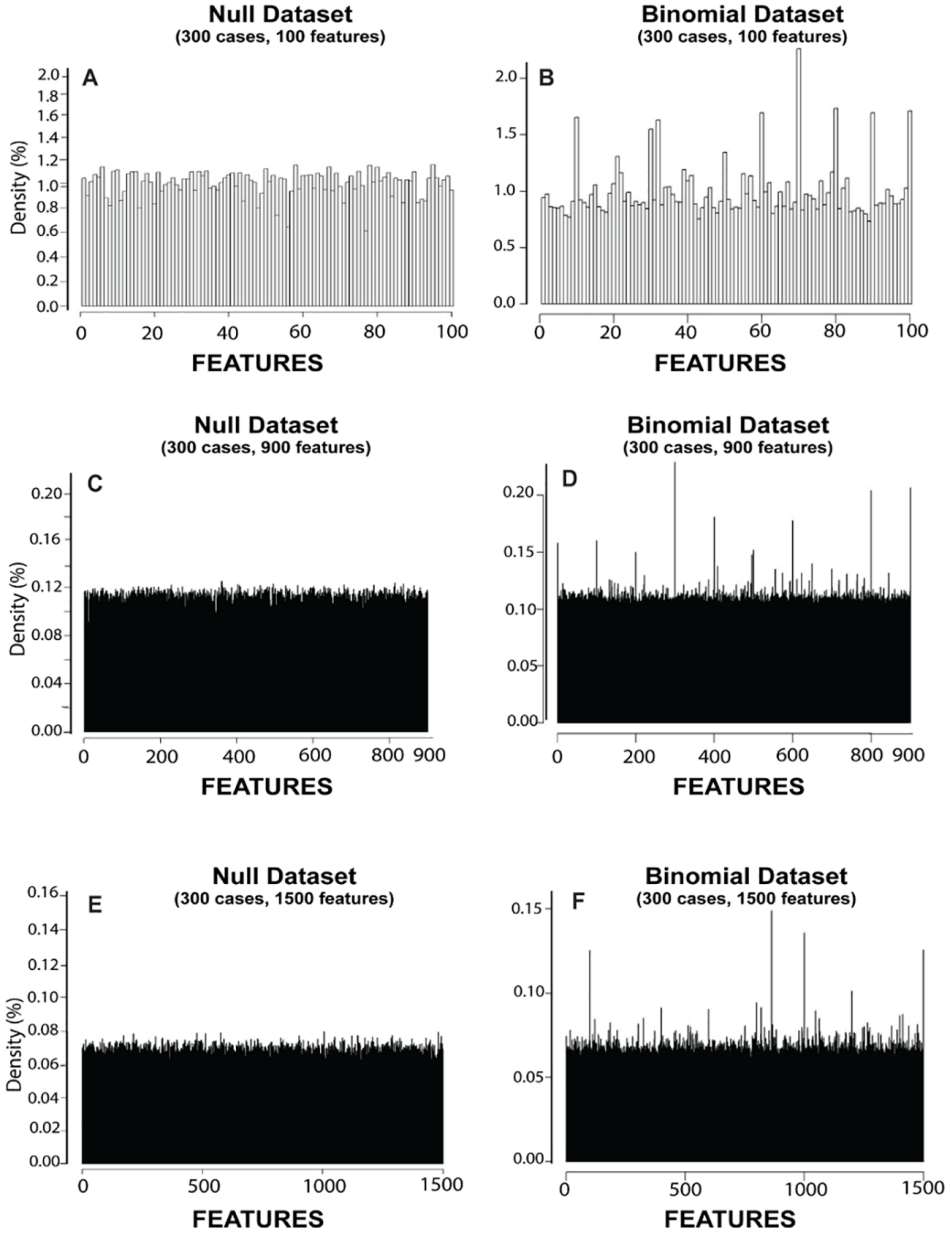
CBDA results on the null and binomial datasets. *Panels A*, *C and E* show the correspondent histograms generated from the CBDA analysis on the three Null datasets. *Panels B*, *D and F* show the correspondent histograms generated from the CBDA analysis on the three Binomial datasets. Panels A and B, C and D, E and F show the combined results of all 12 experiments using the MSE metric.

Fig 4 summarizes the CBDA results on the Null and the Binomial datasets and compares those to the regularized linear model with knockoff filtering. The goal in this case is to validate the CBDA protocol when no signal is present in the data.

For the best accuracy in the comparison, we used all the 9,000 CBDA samples, ranking the top 1,000 predictions (this is equivalent to the combination 16 in Fig 3). Each histogram in Fig 4 combines the results of all 12 experiments (using the MSE metric). A common outcome from the Null datasets analyses is the inconsistent sets of top 15 features returned across different experiments. Thus, by combining the experiments together, the histograms for the Null datasets show uniform distributions of the features selected (flat distribution with no spikes). This is consistent with the fact that the Null datasets have no signal (see the first column of Fig 4). This result is consistent throughout the three Null datasets, with the correspondent constant densities possibly a function of the CBDA specifications (i.e., FSR) and dataset sizes (i.e., number of features). Specifically, the MSE histograms of the combined experiments return flat distributions at ~1%, 0.12% and 0.07%, respectively for the 3 Null datasets. A similar threshold can be seen in the second column of Fig 4, where we show the correspondent histograms generated from the CBDA analysis on the three Binomial datasets. Anything above these thresholds can be considered a signal, or a "true positive". This suggests that a hard threshold for false discovery rates in the CBDA protocol can be computed theoretically. The complete set of results for the histograms in Fig 4 are shown in S3 Text **(Supplementary Materials)**. A total of 234,000 jobs have been performed on the Pipeline Cranium server, resulting from the analysis of three null datasets, from 8 to 10 experiments and 9,000 CBDA samples for each experiment.

**Fig 5:**
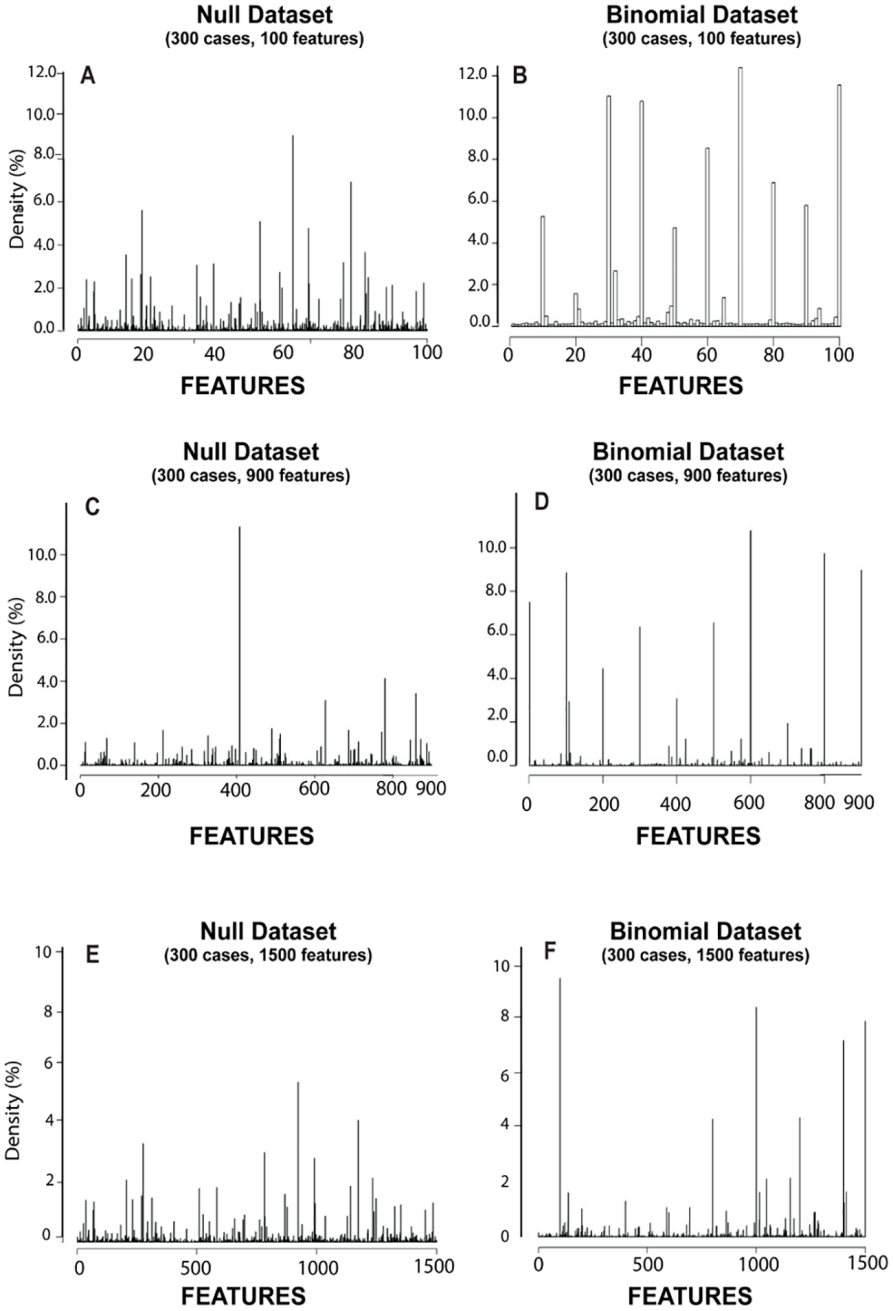
Knockoff filtering of null vs binomial data. *Panels A*, *C and E* show the correspondent histograms generated from the Knockoff Filter algorithm on the three Null datasets. *Panels B*, *D and F* show the correspondent histograms generated from the Knockoff Filter algorithm on the three Binomial datasets. Panels A and B, C and D, E and F show the combined results of all 12 experiments using the MSE metric.

Fig 5 shows combined parallel results using the Null and Binomial datasets, based on a regularized linear model with knockoff filtering. While the knockoff (KO) filter performs quite well in the Binomial datasets (as expected), many false positives are present in the Null datasets analyses. We use a 5% false discovery rate (FDR) as an input for the Knockoff filter algorithm, so anything above 5% in the histograms should be considered a false positive. Despite the fact that the Null dataset includes no real signal, the KO filter algorithm returns few spikes (i.e., false positives) above the hypothetical threshold imposed in the KO filter call for false discovery rate, i.e. 5%. The CBDA returned uniform distributions for the Null datasets, suggesting a more robust overall filtering of false positive feature discoveries. Because of the large scale of the simulations, the testing protocol is mostly invariant of the proportion of samples that contain a subset of the relevant features, if any. Certainly the probability of salient features being chosen as part of each iterative subsample depends on the size of the feature space.

### CBDA robustness

We investigated the robustness of the CBDA algorithm by running the protocol 10 times on the same synthetic dataset (i.e., Binomial Dataset 3). The Binomial dataset 3 has 300 cases and 900 features, where the "true" features are 1, 100, 200, 300, 400, 500, 600, 700, 800 and 900. For each replication, we listed the top 10 features selected by the CBDA, using the two performance metrics available, namely Accuracy and MSE. Each replication has 9,000 samples generated by the CBDA protocol on the large dataset (similarly to our previous set of experiments shown in Fig 3). We use the specifications of experiment 6, where we set the missing values to 0, the CSR to the [60%-80%] range and the FSR to the [15%-30%] range. Overall, features 300, 800, 900, 400, 100, 600, 500, 1 and 700 have been consistently selected within the top 10 features across the 10 replications using the MSE metric (see S1 Table for details and https://github.com/SOCR/CBDA for the complete set of results across the 10 replications). We performed the same experimental design (i.e., CSR, FSR and 10 replications) on the other Binomial dataset obtaining similar results in terms of a consistent selection of top true features (see S1 Table for details). These results confirm the robustness of the CBDA protocol on simulated data.

### CBDA application to ADNI clinical data

We performed four different experiments, each one with 9,000 independent samples, using the ADNI dataset, see Table 1 for details on the ADNI Archive and S2 Table for details on the list of features. The Feature Sampling Ranges used are [5%,15%] and [15%,30%], since the range [1%-5%] was not viable, given the number of features available for analysis (i.e., 64). The Case Sampling Ranges were [30%-60%] and [60%-80%], respectively. We chose to hold off 20% of the cases (i.e., *α* = 20%) for the balanced validation set. Throughout the analysis, imputation (see [24] for details), normalization (see [25] for details) and balancing (see [26] for details) was performed.

As described in the Methods section, the ADNI dataset consists of 2,500 participants representing three clinical phenotypes. These three cohorts represent EO-AD (Early Onset Alzheimer Disease, N_1_ = 406), Normal (N_2_ = 747) and EO-MCI (Early Onset Mild Cognitive Impairment, N_3_ = 1,347).

CBDA protocol ranked the top 1,000 predictions out of the 9,000 learning samples (i.e., Combination 16 in Fig 3) and consistently returned across the 4 experiments the following top 10 features (listed in order of importance): CD Global, weight [kg], sex, age, Right cingulate gyrus, FAQ Total, Left gyrus rectus, Right putamen, cerebellum and Left middle orbitofrontal gyrus. We used these 10 features as input features for a final SuperLearner analysis on the validation set, obtaining a 95% confidence interval of [87.67%, 93%] for accuracy. We didn’t enforce stopping criteria in this case, assuming that a predictive model with only 10 features would be an adequate and parsimonious set of specifications. The confidence intervals of the sensitivity (85% and 94%) and specificity (91% and 98%) to predict the participant phenotype, across the 3 cohorts, are shown on Table 4. The Synthetic Minority Oversampling Technique (SMOTE) rebalancing approach (see [26] and S2 Text for details) significantly improved the accuracy, sensitivity and specificity of our predictions for each of the three cohorts. The complete set of results is shown at the following link: https://github.com/SOCR/CBDA.

**Table 4:** CBDA multinomial classification results on the ADNI dataset. Confusion Matrix and Statistics.

	Reference		
Prediction	*AD*	*MCI*	*Normal*
***AD***	69	17	1
***MCI***	12	243	8
***Normal***	0	9	140
			
Overall Statistics			
Accuracy	0.9058 [95% CI = (0.8767,0.93)		
No Information Rate	0.5391		
p-value [Acc>NIR]	<2e-16		
Kappa	0.8426		
McNemar’s Test p-value	0.589		
			
	**Statistics by Diagnostic Class**		
	**AD**	**MCI**	**Normal**
Sensitivity	0.8519	0.9033	0.9396
Specificity	0.9569	0.913	0.9743
Positive Pred Value	0.7931	0.924	0.9396
Negative Pred Value	0.9709	0.8898	0.9743
Prevalence	0.1623	0.5391	0.2986
Detection Rate	0.1383	0.487	0.2806
Detection Prevalence	0.1743	0.5271	0.2986
Balanced Accuracy	0.9044	0.9082	0.9569

For completeness, we also tested the CBDA protocol with a binary outcome (i.e., AD patients vs. asymptomatic Normal controls) to compare these results against previous studies [31–33]. These results are shown in S3 Table. We used the top 10 features selected by the CBDA protocol and the CBDA results are similar to our previous study in terms of accuracy and other performance metrics. The CBDA approach presented here improves the classification step by considering a multinomial outcome, e.g., AD vs. Normal vs. MCI. The hardest classification task is in fact in separating the AD from the MCI patients.

## Conclusions and discussion

Many challenges and opportunities are embedded in the Big Data revolution. Magnitude, complexity, incongruency and heterogeneity are just some of the attributes of the massive dynamic and spatio-temporal data that we are trying to collect, harmonize, interrogate and mine for actionable knowledge. A divide-and-conquer approach is reasonable to tackle each and every challenge and transform it into an opportunity for developing new tools and foster scientific discovery. The massive amount of information embedded in Big Data is by no means complete and coherent, and one key challenge is to reconstruct a mechanistic explanation out of sparse large datasets. A trade off exists between accuracy of our predictive analytics and the speed at which this hidden actionable knowledge can be acquired. A reasonable compromise will entail an efficient computational platform that can handle the interrogation of chunks of the Big Data, ensuring that the reconstruction steps converge given certain properties of the data processed and the algorithmic framework of the learning stage.

The compressive big data analytics method takes a divide-and-conquer approach utilizing ideas from Compressive Sensing (CS) and Signal Processing (SP), e.g., randomized undersampling, to iteratively sample, estimate and infer using an adaptive error correction/control [6, 7, 34–36]. The CBDA approach has several parallels with CS strategies. Compressive Sensing aims at using an observation matrix to capture a signal, and reconstruct the signal by sparsely captured information. In our CBDA approach, we also define an observation matrix, or we can say a dictionary (feature selection) matric to observe Big dataset, we keep few useful information just as CS reserves sparse information, but our goal here is to do prediction, not reconstruction. We use an ensemble algortihm (i.e., SuperLearner) to combine as many machine learning, classification, statistical modeling tools to ensure the best predictive model generation given the available data.

We tested our first generation CBDA protocol on both synthetically generated and real datasets. Our results on synthetically generated datasets are encouraging. Even with random undersampling rates (~1%-5%), the CBDA protocol can identify most of the true features. This is relevant since a predictive model might not need necessarily the whole set of true features to generate very accurate predictions. Throughout our validation tests, knockoff filtering results were generally better than the CBDA protocol where signal was present, suggesting that CBDA has a more robust framework when dealing with very noisy datasets, or where the signal is non-existent.

The CBDA classification results on the Alzheimer Disease Neuro Imaging (ADNI) case-study provide empirical evidence of effective prediction of clinical outcomes, especially if compared to previous studies in the field [31–33]. Korolev *et al*. [31] developed a multivariate prognostic model for predicting MCI-to-dementia progression at the individual patient level over a 3-year period. Their best performing model incorporated a combination of cognitive/functional markers and morphometric MRI measures and predicted progression with 80% accuracy (83% sensitivity, 76% specificity, AUC = 0.87). Another study by Prestia *et al*. [32] analyzed the ADNI data and reported a combination of biomarkers (i.e., Aβ42 concentrations and hippocampal volumes) to identify prodromal AD (i.e., MCI patients progressing to AD). Their sensitivity was ~79%, with accuracies up to 73%. Another approach was to predict clinical scores from individual MRI scans. Stonnington *et al*. [33] used relevance vector regression (RVR) to predict clinical scores from individual scans, obtaining very high correlations. The RVR results indicated correlation between observed and predictive clinical outcomes (e.g., AVLT, MMSE, and ADAS-Cog) in the range 0.4–0.65, which may be sufficient for clinical diagnoses or prediction of Alzheimer’s progression over time.

The CBDA diagnostic prediction reached average accuracy of ~90% performing multinomial classification on Normal, AD and MCI patients, in a cohort of 2,500 individuals. This substantive prediction performance improvement may be due to (1) the larger number of subjects included in the CBDA study, (2) the flexible and extensive ensemble of machine learning, classification algorithms and model-free methods built into the SuperLearner, or (3) the compressive sensing strategy of repeated stochastical (re)sampling of the data and the specific CBDA inference-aggregation protocol. These results provide evidence of the effectiveness of CBDA to deal with complex and heterogeneous real data application. Further studies will examine the theoretical CBDA properties (e.g., convergence, asymptotic trends, upper error bounds, etc.) as well as the performance of the CBDA technique on larger biomedical datasets where issues of sparsity, incongruence, heterogeneity and missingness may be amplified by increasing the study population and/or the feature characteristics.

The CBDA protocol is designed and built based on open-source/open-science principles where the scientific community can independently test, validate and expand on this first generation technology (https://github.com/SOCR/CBDA). A CBDA Github repository is available to replicate our results as well as to apply the CBDA protocol to other datasets. The current CBDA protocol validation will also be expanded to comprise other synthetically generated datasets with known true features using more complex models for specifying the underlying signal in the simulated data. Two open-source implementations of the CBDA protocol are available—platform-agnostic stand-alone R package (https://cran.r-project.org/package=CBDA) as well as a reproducible pipeline graphical workflow (wrapper of the R-package).

There are several limitations for the protocol. Future expansion and enhancement is required to maximize its functionality. For instance, the protocol may be scaled up, to efficiently handle datasets of millions of cases and thousands of features, or tested on synthetic datasets simulated with more complex and sophisticated models. We are now working on both challenges recasting the existing protocol to handle larger datasets. An updated workflow will be designed to implement *Steps 1–5* of the CBDA protocol offline, where thousands of small matrices will be generated remotely according to the CSR and FSR specs. Then, *Steps 6–8* of the existing workflow will be tested by loading each small matrix independently (instead of loading the entire Big Data). It will need to be investigated how the number of iterations needed for the CBDA protocol to converge will be affected when low CSR and FSR specifications are used in larger datasets. Similarly to more recent studies in CS [34–36], we are also exploring the CBDA mathematical properties, e.g., condutions that may guarantee CBDA convergence.

## Supporting information

S1 Text Pseudocode of the CBDA protocol. Pseudocode of the CBDA-SL algorithm as implemented purely in R as well as via the LONI graphical pipeline workflow environment.
(DOCX)

S2 Text SuperLearner and the ensemble algorithms used. Details about data imputation, normalization, rebalancing, as well as model-based and model-free analytics, including Linear Models, Elastic Net, Random Forest, SVM, BartMachine, SuperLearner, and Knockoff controlled variable selection.
(DOCX)

S3 Text CBDA complete set of results. Additional Results.
(DOCX)

S4 Text Mathematical formulation of CBDA. (DOCX)

S1 Table CBDA robustness. (DOCX)

S2 Table ADNI features. (DOCX)

S3 Table ADNI results binary outcome. (DOCX)

## References

[1] Agarwal R, Dhar V. Editorial—Big Data, Data Science, and Analytics: The Opportunity and Challenge for IS Research. Information Systems Research. 2014;25(3):443–8.

[2] Familiar B, Barnes J. Advanced Analytics Using Machine Learning and R Business in Real-Time Using Azure IoT and Cortana Intelligence Suite: Springer; 2017 p. 351–96.

[3] Babu SK, Vasavi S, Nagarjuna K, editors. Framework for Predictive Analytics as a Service Using Ensemble Model. Advance Computing Conference (IACC), 2017 IEEE 7th International; 2017: IEEE.

[4] Slavakis K, Giannakis GB, Mateos G. Modeling and optimization for big data analytics:(statistical) learning tools for our era of data deluge. IEEE Signal Processing Magazine. 2014;31(5):18–31.

[5] Wilkinson MD, Dumontier M, Aalbersberg IJ, Appleton G, Axton M, Baak A, et al The FAIR Guiding Principles for scientific data management and stewardship. Scientific data. 2016;3:160018 10.1038/sdata.2016.18 26978244 PMC4792175

[6] Barber RF, Candes EJ. Controlling the False Discovery Rate Via Knockoffs. Ann Stat. 2015;43(5):2055–85. 10.1214/15-Aos1337

[7] Rauhut H. Compressive sensing and structured random matrices. Theoretical foundations and numerical methods for sparse recovery. 2010;9:1–92.

[8] Candes E, Fan Y, Janson L, Lv J. Panning for gold:‘model‐X’knockoffs for high dimensional controlled variable selection. Journal of the Royal Statistical Society: Series B (Statistical Methodology). 2018.

[9] Strobl C, Malley J, Tutz G. An introduction to recursive partitioning: rationale, application, and characteristics of classification and regression trees, bagging, and random forests. Psychological methods. 2009;14(4):323 10.1037/a0016973 19968396 PMC2927982

[10] Hothorn T, Lausen B. Bundling classifiers by bagging trees. Computational Statistics & Data Analysis. 2005;49(4):1068–78.

[11] Polley E, LeDell E, Kennedy C, Lendle S, van der Laan M. Package ‘SuperLearner’. CRAN; 2017.

[12] van der Laan MJ, Polley EC, Hubbard AE. Super learner. Stat Appl Genet Mol Biol. 2007;6: 25 10.2202/1544-6115.1309 . 17910531

[13] Chang C-C, Lin C-J. LIBSVM: A library for support vector machines. ACM Trans Intell Syst Technol. 2011;2(3):1–27.

[14] Chipman HA, George EI, McCulloch RE. BART: Bayesian additive regression trees. The Annals of Applied Statistics. 2010;4(1):266–98.

[15] Friedman J, Hastie T, Simon N, Tibshirani R. Lasso and Elastic-Net Regularized Generalized Linear Models. R-package version 2.0–5. 2016.

[16] Hastie TJ, Tibshirani RJ. Generalized additive models: CRC press; 1990. 10.1177/096228029500400302 8548102

[17] Hearst MA, Dumais ST, Osuna E, Platt J, Scholkopf B. Support vector machines. IEEE Intelligent Systems and their applications. 1998;13(4):18–28.

[18] Hornik K, Meyer D, Karatzoglou A. Support vector machines in R. Journal of statistical software. 2006;15(9):1–28.

[19] Kapelner A, Bleich J. bartmachine: Machine learning with bayesian additive regression trees. arXiv preprint arXiv:13122171. 2013.

[20] McCullagh P, Nelder J. Generalised linear models II. London: Chapman and Hall; 1989.

[21] Zou H, Hastie T. Regularization and variable selection via the elastic net. Journal of the Royal Statistical Society: Series B (Statistical Methodology). 2005;67(2):301–20.

[22] Dinov ID, Petrosyan P, Liu Z, Eggert P, Hobel S, Vespa P, et al High-throughput neuroimaging-genetics computational infrastructure. Frontiers in Neuroinformatics. 2014;8. 10.3389/fninf.2014.00041 PMC4005931 24795619

[23] Boehmke BC. Data Wrangling with R: Springer; 2016.

[24] Stekhoven DJ, Buhlmann P. MissForest—non-parametric missing value imputation for mixed-type data. Bioinformatics. 2012;28(1):112–8. 10.1093/bioinformatics/btr597 . 22039212

[25] Becker RA, Chambers JM, Wilks AR. The new S language: a programming environment for data analysis and graphics. Pacific Grove, Calif: Wadsworth & Brooks/Cole Advanced Books & Software; 1988 xvii, 702 p. p.

[26] Chawla NV, Bowyer KW, Hall LO, Kegelmeyer WP. SMOTE: Synthetic minority over-sampling technique. J Artif Intell Res. 2002;16:321–57.

[27] Moon SW, Dinov ID, Hobel S, Zamanyan A, Choi YC, Shi R, et al Structural Brain Changes in Early-Onset Alzheimer’s Disease Subjects Using the LONI Pipeline Environment. J Neuroimaging. 2015;25(5):728–37. 10.1111/jon.12252 . 25940587 PMC4537660

[28] Dinov ID. Methodological challenges and analytic opportunities for modeling and interpreting Big Healthcare Data. Gigascience. 2016;5:12 10.1186/s13742-016-0117-6 . 26918190 PMC4766610

[29] Moon SW, Dinov ID, Kim J, Zamanyan A, Hobel S, Thompson PM, et al Structural Neuroimaging Genetics Interactions in Alzheimer’s Disease. J Alzheimers Dis. 2015;48(4):1051–63. 26444770 10.3233/JAD-150335 PMC4730943

[30] Jack CR Jr., Bernstein MA, Fox NC, Thompson P, Alexander G, Harvey D, et al The Alzheimer’s Disease Neuroimaging Initiative (ADNI): MRI methods. J Magn Reson Imaging. 2008;27(4):685–91. 10.1002/jmri.21049 . 18302232 PMC2544629

[31] Korolev IO, Symonds LL, Bozoki AC, Initiative AsDN. Predicting progression from mild cognitive impairment to Alzheimer’s dementia using clinical, MRI, and plasma biomarkers via probabilistic pattern classification. PloS one. 2016;11(2):e0138866 10.1371/journal.pone.0138866 26901338 PMC4762666

[32] Prestia A, Caroli A, Herholz K, Reiman E, Chen K, Jagust WJ, et al Diagnostic accuracy of markers for prodromal Alzheimer’s disease in independent clinical series. Alzheimer’s & dementia: the journal of the Alzheimer’s Association. 2013;9(6):677–86. 10.1016/j.jalz.2012.09.016 23375562 PMC4058442

[33] Stonnington CM, Chu C, Klöppel S, Jack CR, Ashburner J, Frackowiak RSJ. Predicting clinical scores from magnetic resonance scans in Alzheimer’s disease. NeuroImage. 2010;51(4):1405–13. 10.1016/j.neuroimage.2010.03.051. 20347044 PMC2871976

[34] Candès EJ, Wakin MB. An introduction to compressive sampling. IEEE Signal Processing Magazine. 2008;25(2):21–30.

[35] Dai W, Milenkovic O. Subspace pursuit for compressive sensing signal reconstruction. IEEE Transactions on Information Theory. 2009;55(5):2230–49.

[36] Foucart S, Rauhut H. A mathematical introduction to compressive sensing: Birkhäuser Basel; 2013.

