# Supplementary material for: Controlled feature selection and compressive big data analytics: Applications to biomedical and health studies
Source: PLoS One. 2018 Aug 30;13(8):e0202674. doi: 10.1371/journal.pone.0202674 (PMC6116997; doi:10.1371/journal.pone.0202674)
Supplement: S1 Text — Pseudocode of the CBDA-SL algorithm as implemented purely in R as well as via the LONI graphical pipeline workflow environment. (DOCX) [file pone.0202674.s001.DOCX]

Controlled Feature Selection and Compressive Big Data Analytics: Applications to Biomedical and Health Studies

Simeone Marino, Jiachen Xu, Yi Zhao, Nina Zhou, Yiwang Zhou, Ivo D. Dinov

**SUPPLEMENTARY INFORMATION**

# S1 Text: Pseudocode of the CBDA protocol

Here we provide the specifics of conducting different experiments [i_exp] testing the CBDA-SL and Knockoff algorithms on the Big Datasets $B=[X,Y]$. Each experiment is uniquely identified by a set of input specifications that are read from an argument file.

Input Specifications = [M ,misValperc,min_FSR,max_FSR,min_CSR,max_CSR], where:

- *M*: number of the Big Data subsets $[X^{j},Y^{j}]$, on which perform Knockoff Filter and CBDA-SL feature mining
- misValperc: % of missing values to introduce in Big Data (used just for testing, to mimic real cases).
- min_FSR: Lower bound for the % of features/columns sampling
- max_FSR: Upper bound for the % of features/columns sampling
- min_CSR: Lower bound for the % of cases/rows sampling
- max_CSR: Upper bound for the % of cases/rows sampling.

Sampling ranges for cases (**CSR** - Cases Sampling Range) and features (**FSR** - Feature Sampling Range) are then defines as follow:

FSR = [min_FSR, max_FSR]

CSR = [min_CSR, max_CSR].

Before each experiment is performed, the necessary data wrangling (e.g., cleaning, harmonization, aggregation and more) is performed on the Big Data to generate 2 sets of data:

1. Data to hold off for validation $[X_{val},Y_{val}]$ (never used for learning/training). We hold off 20% of the subjects for validation and confusion matrix construction. This fraction to hold off can be set as an input as well. A fixed seed warrants the same selection $[X_{val},Y_{val}]$ for the validation set across all the instances of each experiment. A fixed seed also ensures reproducibility of our results.
2. Data to be sampled for learning/training by the CBDA-SL and Knockoff algorithms $[X_{temp},Y_{temp}]$.

Here we refer to X-datasets as datasets of features, while Y-datasets are datasets of outcomes (binomial/multinomial). If Big Data is within the order of GBs, alternative strategies can be implemented to avoid loading large datasets before launching the CBDA-SL and Knockoff algorithms. For example, we could generate all the M data subsets offline and save them in separate RData workspaces, and then load the appropriate ones for each of the M job.

Also, the set of input specifications should be carefully designed so that, given certain constraints of the algorithms used, all the conditions are fulfilled, such as a minimum number of features selected to run the CBDA protocol or that the number of cases should be greater than the number of features (e.g., the latter constraints is for the current knockoff filter algorithm implementation, see below).

For each $j=1:M$, we load the Big Data and generate a $X^{j}$, $X_{val}^{j}$ and a $Y^{j}$ by sampling $X_{temp}$, $X_{val}$ and $Y_{temp}$, respectively. FSR and SSR specifications are used to generate $k_{j}$ and $n_{j}$, which represent the subsets of features/columns and subjects/rows, respectively. $k_{j}$ and $n_{j}$ are then used to generate the matrices $X^{j}$ , $X_{val}^{j}$ and $Y^{j}$ to be passed to the Knockoff Filter and CBDA-SL algorithms. We can enforce balanced $Y_{val}$ , $X_{val}^{j}$ , $X^{j}$ and $Y^{j}$ (i.e., equal number of cases for each outcome category). The package SMOTE in R automatically balanced the datasets if the discrepancy is greater than a threshold (e.g., 50%).

$X^{j}$ and $X_{val}^{j}$ are then imputed and normalized. Imputation and normalization can also be done on the Big Data, before sampling is performed. A trade-off in computation time can be investigated; however, it should be significant only when the original dataset is very large and the FSR/CSR are not small. If FSR/CSR are very small (~5%), an inline/real-time imputation might be more efficient. For imputation we use the function missForest, with max # of iterations ~5-10 for convergence (see the description of the different algorithms for details).

## Pseudocode of the CBDA-SL algorithm as implemented in the LONI pipeline workflow

for (row i=1: i_exp) [# experiments 5-50] {

for (j=1:M,M~5K-50K) [M is j_global in the workflow]

{

Knockoff.filter (j)( $X^{j},Y^{j},...$) SuperLearner (j)( $X^{j},Y^{j},...$)

Save results in RData workspaces [..light_j.RData]

}

}

Knockoff.filter(j)

{

1. The knockoff filter is a procedure for controlling the false discovery rate (FDR) when performing variable selection. The version implemented here has the constraint that $n>k$ (# of subjects > # of features). Thus, particular attention is needed in building the set of input specification.
2. Returns features selected after passing matrix $X^{j}$ and outcome $Y^{j}$.

}

SuperLearner (j)

{

1. Combines many learning/optimization algorithms [10-30 different algorithms]
2. Performs Cross Validation for each one (~ 10 CV folds)
3. Generate predictions and 2 metrics to later rank them: Mean Square Error [MSE_j] and Accuracy [Accuracy_j] comparing to $Y_{val}$

}

Save results in RData workspaces [..light_j.RData]

{

The workspace j generated after performing the operations above is too big to be saved as is. Thus, the SLj object (~50-100Mb) is deleted, and the workspace j saved for post-optimization purposes.

}

The workflow is now parallelized; each j is an independent job. Multiple experiments can be combined in a single pipeline workflow. However, we are constrained by the total number of jobs that can be submitted in a single workflow on Cranium (3K). The latest version of the pipeline workflow combines 3 modules of 3K jobs each for each experiment, for a total of 9K jobs for each experiment.

**Post Optimization process**

**1st Step - Workspaces consolidation and metrics**

for (i=1: i_exp) [# experiments 5-50]

{ for (j=1:M) { Load ..CBDA…._j.RData }

1. The consolidated array of the metrics MSE and Accuracy are generated, sorted and a subset of top-ranked jobs are selected (e.g., in **Fig 2** and **Table 5** we list as 100, 200, 500 and 1,000 Top-Ranked [j] jobs). The selection of the top jobs is a parameter in the code and can be expanded or trimmed.
2. To avoid stack overflow errors, several named objects are deleted (i.e., j=1,2,...,M $\to$ MSE_j and Accuracy_j are deleted)
3. The consolidated Rdata workspace is saved (~5-10 Mb).
4. The single j Rdata workspaces are then deleted, resulting in one complete Rdata workspace with all the results (out of 9,000).

}

**2nd Step - Results display**

An R Markdown file generates results for single or multiple experiments pertaining the analysis of a specific Big Data. The distribution of the features ($f_{i}$) suggested by the top-ranked predictions based on the 2 metrics (e.g., lowest MSE, highest Accuracy) is plotted in a density histogram for the KO, MSE and Accuracy, to possibly display spikes/signals$\to$ Feature Mining Tables are also generated with information on frequency of occurrences and densities of each of the features in the top 15 features. We choose the top 15 for ease of illustration only. The top features selected are also displayed by label.

**MSE and Accuracy: Ranking and Feature Mining**

Feature Mining can be performed in 3 ways:

1. By single experiment
2. Combining all the experiments
3. Combining subsets of experiments

The following points are the same across 1, 2 and 3 above for the CBDA-SuperLearner predictions.

1. Calculate Mean Square Error between data to be predicted and the CBDA-SL predictions - MSE_j
2. Calculate the Accuracy between data to be predicted and the CBDA-SL predictions - Accuracy_j (we use the confusion matrix tool).
3. Select the TOP MSEs and Accuracy (TOP=~100-1,000)
4. Extract the features $f_{i}$ used for each of the TOP MSEs and Accuracies and build an histogram
5. Merge all the 9,000 features selected by each knockoff.filter(j) and build an histogram
6. Spikes in the histograms will indicate the features that are most common among the best predictions $\to$ Feature Mining

We tested the CBDA-SL and the Knockoff filter on ad hoc datasets to determine their FDRs and LOD (level of detection) of TP (true positive) and FP (False Positive) features. We investigated the rate of convergence of the algorithms under different specs (primarily the # of jobs needed and top-ranked predictions). Each job takes ~2-10 minutes. Using cross-validation with SuperLearner adds ~20-100 minutes per job.
