# Supplementary material for: Controlled feature selection and compressive big data analytics: Applications to biomedical and health studies
Source: PLoS One. 2018 Aug 30;13(8):e0202674. doi: 10.1371/journal.pone.0202674 (PMC6116997; doi:10.1371/journal.pone.0202674)
Supplement: S2 Text — Details about data imputation, normalization, rebalancing, as well as model-based and model-free analytics, including Linear Models, Elastic Net, Random Forest, SVM, BartMachine, SuperLearner, and Knockoff controlled variable selection. (DOCX) [file pone.0202674.s002.DOCX]

Controlled Feature Selection and Compressive Big Data Analytics: Applications to Biomedical and Health Studies

Simeone Marino, Jiachen Xu, Yi Zhao, Nina Zhou, Yiwang Zhou, Ivo D. Dinov

**SUPPLEMENTARY INFORMATION**

# S2 Text: SuperLearner [11] and relevant ensemble algorithms

## Imputation and Normalization

We use a nonparametric missing value imputation strategy based off Random Forest. We used the R function 'missForest' to impute missing values because we typically have cases of mixed-type data. It can be used to impute continuous and/or categorical data including complex interactions and nonlinear relations. It yields an out-of-bag (OOB) imputation error estimate. Moreover, it can be run parallel to save computation time, see [24] for more details. We use a standard normalization technique, see [25] for details.

## Re-balancing - SMOTE

Synthetic Minority Oversampling Technique (SMOTE) was used as a rebalancing approach. SMOTE works by creating synthetic samples from the minor class instead of creating copies. The algorithm selects two or more similar instances (using a distance measure) and perturbing an instance one attribute at a time by a random amount within the difference to the neighboring instances, see [26] for details.

**SL.library: Algorithms description** [some text here is needed for the SuperLearner Function]

**Notations:**

- X: a predictor column-joined.
- Y: the response variable.
- k: the number of predictors.
- n: the number of observations.

## Generalized linear model (GLM)

GLM is a fitting generalized linear model, specified by giving a symbolic description of the linear predictor and a description of the error distribution. This algorithm could be used on both categorical or discrete variables and continuous variables. In the meantime, instead of assuming that the responses confirm to normal distribution, the response variables could come from any exponential families.

**Advantages:**

- This model could extend the linear model to problems in which the response is categorical or discrete rather than a continuous numeric variable. GLM does not assume a linear relationship between the dependent variables and the independent variables, but it does assume linear relationship between the transformed response in terms of the link function and the explanatory variables.
- In the Generalized Linear Model (GLM), the response variable may not be normally distributed. For instance, the response variable may be binary (logistic model). GLM addresses these cases - e.g., GLM logit and probit models are GLM special cases appropriate for dichotomous or polytomous variables.

**Assumptions:**

The data $Y_{1},...,Y_{N}$ are independently distributed and are assumed from an exponential family.

**Notations:**

$\beta^{\tau}=(\alpha,\beta_{1},...,\beta_{k})$: The parameters of the linear predictor. GLM are typically fit to data by the method of **maximum likelihood**, using **iteratively weighted least squares procedure**. Assume that the maximum-likelihood estimates of the regression parameters are $\hat{\beta}^{\tau}=(\hat{\alpha},\hat{\beta}_{1},...,\hat{\beta}_{k})$:

**Model components:**

*A random component*: The random component specifies the conditional distribution of the response variable, Y, given the predictors. And $\mu(x)$ denotes the expectation of Y. The conditional distributions are from an exponential family.

*A linear predictor*

$\eta(x)=\alpha+\beta_{1}x_{1}+...+\beta_{k}x_{k}$: $\eta(x)$ is the value of the linear predictor. And $\overset{^}{\eta}(x)$ represents the estimated value of the linear predictor. Hence, the GLM models is shown as followed:

$$\overset{^}{\eta}\left( x \right)=\hat{\alpha}+\hat{\beta}_{1}x_{1}+...+\hat{\beta}_{k}x_{k}.$$

*A link function*: $g[\mu(x)]=\eta(x)$, which enables the connection of the predictor structural component to the response variable, since in some case $\eta(x)$ may take on any value in $(-\infty,+\infty)$, whereas the mean of a binary random variable must be in the interval (0,1). The link function translates from the scale of the mean response to the scale of the linear predictor.

$\mu(x)$ is the mean of a conditional response distribution at a given point in the covariate space and $\eta(x)$ is the result predicted from the linear predictor. The link function is important as it specifies the distribution of the response variable- e.g., identity link function for normal and non-identity link function for non-normally distributed responses.

The choice of the link function is important and should be based on:

1. Knowledge of the response distribution,
2. Theoretical process assumptions,
3. Empirical fit to the data.

In R, the glm function includes a family-generator function, which could match link functions to five standard exponential families automatically. Each family has its own canonical link, which is used by default if a link is not given explicitly.

| Family | Default link | Range of y | Var(y$\vert\eta(x)$) |
| --- | --- | --- | --- |
| gaussian | identity | $(-\infty,+\infty)$ | $\phi$ |
| binomial | logit | $\frac{(0,1,...,N)}{N}$ | $\frac{\mu(1-\mu)}{N}$ |
| poisson | log | 0,1,2,... | $\mu$ |
| Gamma | Inverse | $(0,+\infty)$ | $\phi\mu^{2}$ |
| inverse.gaussian | $\frac{1}{\mu^{2}}$ | $(0,+\infty)$ | $\phi\mu^{3}$ |

**Statistical Analysis:**

As shown above the estimated value of the linear predictor is $\overset{^}{\eta}(x)=\hat{\alpha}+\hat{\beta}_{1}x_{1}+...+\hat{\beta}_{k}x_{k}$

- The estimated mean of the response is $\overset{^}{\mu}\left( x \right)=g^{-1}\left[ \overset{^}{\eta}\left( x \right) \right]=g^{-1}[\hat{\alpha}+\hat{\beta}_{1}x_{1}+...+\hat{\beta}_{k}x_{k}]$, because of the link function $g[ \mu(x)]=\eta(x)$.
- The variance of the distribution is $var(y|\eta(x))=\phi\times v[\mu(x)]$, $\phi$ is the positive dispersion parameter related to the variance of the exponential family. For the binomial and Poisson distributions, the dispersion parameter equal to 1, for Gaussian data, the parameters could be replaced by $\sigma^{2}$, see [37] for details.

## A generalized linear model with lasso or elastic net regularization--GLMNET

GLMNET is a model, which fit a generalized linear model via penalized maximum likelihood. The regularization path is computed for the **LASSO** or **elastic net** penalty at a grid of values for the regularization parameter lambda. This model can deal with all shapes of data, including vary large sparse data matrices. Fits linear, logistic and multinomial, Poisson, and Cox regression models.

$Model component$: The GLM part: the GLM functions for different families are different.

For "Gaussian" family is

$$\frac{1}{2}\times\frac{1}{(observations)}RSS.$$

For the other models are

$$\frac{(-loglik)}{(observations)}.$$

$The penalty is denoted as$:

$$penalty=\frac{1-\alpha}{2}\parallel\beta\parallel_{2}^{2}+\alpha\parallel\beta\parallel_{1}$$

when $\alpha=1$, the penalty becomes the lasso penalty,

when $\alpha=0$, the penalty becomes the ridge penalty.

$Objective functions$:

For "Gaussian" family is: $\frac{1}{2}\times\frac{1}{\left( observations \right)}RSS+\lambda\times penalty.$

For the other models are: $\frac{-loglik}{(observations)}+\lambda\times penalty.$

The shrinkage parameter: The value of $\lambda$ has direct relationship with the final result. For instance, in ridge regression, $\lambda$ controls the size of the coefficients and the amount of regularization.

If $\lambda\downarrow0$, we could obtain the least squares solutions.

If $\lambda\uparrow\infty$, the estimate parameters we get could tend to 0.

In R, the default value of $\lambda$ is a sequence of number. Systems will choose 100 different $lambda$ and the default number of the smallest value of the $\lambda$ depends on the sample size relative to the number of the variables. If k>n, the default is 0.0001, on contrast, the default is 0.01.

$Penalty$:

$LASSO Regularization$. When $\alpha=1$, the penalty is the lasso penalty, the objective function is

$$\beta=argmin_{\beta}\sum_{i=1}^{n} (y_{i}-\beta_{0}-\sum_{j=1}^{k} x_{ij}\beta_{j})^{2}+\lambda\parallel\beta\parallel_{1}.$$

Advantage: Owing to the nature of the $l_{1}$-penalty, the lasso could both continuous shrinkage and automatic variable selection simultaneously.

Limitation:

- When the $k>n$, the lasso selects at most n variables before it saturates, because of the convex optimization problem. Moreover, the lasso is not well defined unless the bound on the $l_{1}$-norm of the coefficients is smaller than a certain value.
- If there is a group of variables among which the pairwise correlations are very high, then the lasso tends to select only one variable from the group and does not care which one is selected.

$Ridge penalty$. When $\alpha=0$, the penalty is the ridge penalty, the function is

$$\beta=argmin_{\beta}\sum_{i=1}^{n} (y_{i}-\beta_{0}-\sum_{j=1}^{k} x_{ij}\beta_{j})^{2}+\frac{\lambda}{2}\parallel\beta\parallel_{2}^{2}.$$

Advantage: As a continuous shrinkage method, ridge regression achieves its better prediction performance through a bias- variance trade off.

Limitation: Since it always keeps all the predictors in the model, this regression method could not produce a parsimonious model.

$Elastic net$. The penalty of elastic net regularized modeling is

$$\frac{1-\alpha}{2}\parallel\beta\parallel_{2}^{2}+\alpha\parallel\beta\parallel_{1},$$

which is a convex combination of the lasso and ridge penalty.

The elastic net simultaneously does automatic variable selection and continuous shrinkage, and it can select groups of correlated variables. The objective function turns to

$$\beta=argmin_{\beta}\sum_{i=1}^{n} (y_{i}-\beta_{0}-\sum_{j=1}^{k} x_{ij}\beta_{j})^{2}+\lambda_{2}\parallel\beta\parallel_{2}^{2}+\lambda_{1}\parallel\beta\parallel_{1}.$$

**Lemma**: Given data set (y, X) and $(\lambda_{1},\lambda_{2})$, define an artificial data set $(y^{*},X^{*})$ by

$$X_{(n+k)\times k}^{*}=(1+\lambda_{2})^{(-1/2)}\left( \begin{matrix} X \\ \sqrt{\lambda_{2}}I \end{matrix} \right),y_{(n+K)}^{*}=\left( \begin{matrix} y \\ 0 \end{matrix} \right).$$

Let $\gamma=\frac{(\lambda_{1})}{\sqrt{1+\lambda_{2}}}$ and $\beta^{*}=\sqrt{1+\lambda_{2}}\beta$. The elastic net criterion can be written as

$$\beta^{*}=argmin_{\beta^{*}}{\parallel y^{*}-X^{*}\beta^{*}\parallel}_{2}^{2}+\gamma\parallel\beta^{*}\parallel_{1}.$$

According to the lemma, this function could be transformed into an equivalent LASSO problem and then be solved.

$Alpha$: Since the value of alpha has directly relationship with the penalty, we choose different value of alpha to expand this algorithm. The default value of alpha is 1, but we apply 0, 0.25, 0.5, 0.75 and 1 to configure different penalty of this algorithm (see [15, 21, 38] for details).

## Classification and regression with Random Forest (Random Forest)

In Random Forest, a tree classifier uses different sub data set to construct a number of trees and form a forest. After a large number of trees are generated, they vote for the most popular class for an input vector and give prediction.

For instance, when generate the $m_{th}$ tree, a random vector $\theta_{m}$ is generated, which is independent of the past random vectors $\theta_{1},\theta_{2},...,\theta_{(m-1)}$ but with the same distribution. And a tree is grown using the training set and $\theta_{m}$, resulting in a classifier $h(x,\theta_{m})$, where x is an input vector. Then each tree casts a unit vote for the most popular class at input x.

Advantages:

- Random forests do not overfit as more trees as added.
- Since each tree is constructed independently, random forests have lower correlation between classifiers.

Some of the key RandomForest parameters include:

**ntree**: the number of trees to grow. This should not be set to too small a number to ensure that every input row gets predicted at least a few times. Significant improvements in classification accuracy have resulted from growing an ensemble of trees and letting them vote for the most popular class.

**mtry**: the number of variables randomly sampled as candidates at each split. The default values are different for classification ($\sqrt{k}$) and regression $\left( \frac{1}{3} \right)$.

**maxnode**: maximum size of terminal nodes. Setting this number larger causes smaller trees to be grown and thus take less time. The default values for classification is 1 and for regression is 5 (see [39, 40] for details).

## Support vector machines (SVM)

SVM is used to train a support vector machine. It can be used to carry out general regression and classification, as well as density- estimation.

Kernel function: In classification, SVM separate the different classes of data by a hyper-plane

$$\langle\phi(x),w\rangle+b=0,$$

where $\phi$ is a mapping of the inputting data into a high-dimensional feature space.

Hence the decision function

$$f(x)=\mathrm{sign}\langle\phi(x),w\rangle+b$$

can be used to represent the result of the classification.

Suppose we have 2 classes, $f(x)=1$, when $x$ belongs to one class OR $f(x)=-1$, when otherwise. It can be shown that the optimal hyper-plane is the one with the maximal margin of the separation between the two classes.

**Maximum margin classifier** We suppose the hyper-lane could be represents as

$\langle\phi(x),w\rangle+b$, hence the distance from a point to this lane is $\frac{f(x)}{\parallel w\parallel}$. The geometrical margin could be represent as $\gamma=y\frac{f(x)}{\parallel w\parallel})$ and the objective function becomes to

$$max\gamma=y\frac{f(x)}{\parallel w\parallel}$$

$$s.t.y_{i}\left( w^{T}\phi\left( x_{i} \right)+b \right)=y_{i}f\left( x_{i} \right)\geq\gamma\parallel w\parallel\left( i=1,2,...,n \right).$$

We could simplify this function to

$$max\gamma=\frac{1}{\parallel w\parallel}$$

$$s.t. y_{i}\left( w^{T}\phi\left( x_{i} \right)+b \right)=y_{i}f\left( x_{i} \right)\geq1\left( i=1,2,...,n \right).$$

since our objective is to find the parameters $w$ and $b$.

This function could then be transferred to convex quadratic programming as followed

$$min\gamma=\frac{1}{2}\parallel w\parallel^{2}$$

$$s.t. y_{i}\left( w^{T}\phi\left( x_{i} \right)+b \right)=y_{i}f\left( x_{i} \right)\geq1\left( i=1,2,...,n \right).$$

Considering the outliers, we could revise this formula to

$$min\gamma=\frac{1}{2}\parallel w\parallel^{2}+\frac{C}{n}\sum_{i=1}^{n} \xi_{i}$$

$$s.t. y_{i}(w^{T}\phi(x_{i})+b)=y_{i}f(x_{i})\geq1-\xi_{i}(i=1,2,...,n)$$

$$\xi_{i}\geq0\left( i=1,2,...,n \right).$$

Adding Lagrange Duality,

$$L\left( w,b,\xi,\alpha,r \right)=\frac{1}{2}\parallel w\parallel^{2}=\frac{C}{n}\sum_{i=1}^{n} \xi_{i}-\sum_{i=1}^{n} \alpha_{i}\left( y_{i}\left( w^{T}\phi\left( x_{I} \right)+b \right)-1+\xi_{i} \right)-\sum_{i=1}^{n} r_{I}\xi_{i}.$$

This problem becomes

$$min_{\left( w,b \right)}max_{\left( \alpha_{i}\geq0 \right)}L\left( w,b,\xi,\alpha,r \right).$$

The dual form of this formulation is

$$max_{\left( \alpha_{i}\geq0 \right)}min_{\left( w,b \right)}L\left( w,b,\xi,\alpha,r \right).$$

Solving the dual form of the formulation through derivation and SMO, we could transfer this formula to

$$max\sum^{i=1} n\alpha_{i}-\frac{1}{2}\sum^{i,j=1} n\alpha_{i}\alpha_{j}y_{i}y_{j}\phi(x_{i})^{T}\phi(x_{j})$$

$$s.t.0\leq\alpha_{i}\leq\frac{C}{n}(i=1,2,...,n)$$

$$\sum_{i=1}^{m} \alpha_{i}y_{i}=0.$$

*Kernel function*: A kernel function returns the inner product between two points in a suitable feature space, thus defining a notion of similarity, with little computational cost even in very high dimensional spaces. The four kernel functions used in R is shown as followed.

| Linear Kernel | $x'x$ |
| --- | --- |
| Polynomial Kernel | $(\gamma x'x+{Coef}_{0})^{deg}$ |
| Radial Basis Kernel | $exp(-\gamma\parallel x'x\parallel^{2})$ |
| Sigmoid Kernel | $tanh$($\gamma x'x+Coef_{0}$) |

Advantages: Since the quadratic programming problem and the final decision function depend only on dot products between patterns, with the help of kernel function, the inside dot product can be represented by a kernel function $k$:

$$k\left( x,x^{'} \right)=\langle\phi\left( x \right),\phi\left( x^{'} \right)\rangle.$$

We could practically work in space of any dimension without any significant additional cost, as the "kernel trick". In the meantime, with kernel function, SVM could generalize the linear algorithm to the non-linear cases. When deal with multi-class classification, SVM applies one-against-one method. Although this suggests a higher number of support vector machines to train the overall CPU time used is less compared to the one-against-all method since the problems are smaller and the SVM optimization problem scales super-linearly.

$Model component$: The default setting for the SCM is c-classification or eps-regression, depending on whether the response variable is a factor or not.

$C-classification$: The dual form of the bound constraint C-SVM formulation is:

$$maxW(\alpha)=\sum_{i=1}^{n} \alpha_{i}-\frac{1}{2}\sum_{i,j=1}^{n} \alpha_{i}\alpha_{j}(y_{i}y_{j}+k(x_{i},x_{i}))$$

$$s.t.0\leq\alpha_{i}\leq\frac{C}{n}(i=1,2,...,n)$$

$$\sum_{i=1}^{n} \alpha_{i}y_{i}=0.$$

Core C-SCV parameters include:

**Type**: svm can be used as a classification machine, as a regression machine, or for novelty detection. Depending of whether y is a factor or not, the default setting for type is C-classification or eps-regression, respectively, but may be overwritten by setting an explicit value.

Valid options are:

- C-classification
- nu-classification
- one-classification (for novelty detection)
- eps-regression
- nu-regression

**Degree**: the parameter needed for kernel of type polynomial. The default value of this is 3.

**Gamma**: the parameter needed for all kernels except linear. The default value of this is $\frac{1}{data}$ dimension.

$\boldsymbol{Coe}\boldsymbol{f}_{\boldsymbol{0}}$: parameter needed for kernels of type polynomial and sigmoid. The default value of this is 0.

**Cost**: cost of constraints violation. The default value of this is 1. A high cost value C will force the SVM to create a complex enough prediction function to misclassify as few training points as possible, while a lower cost parameter will lead to simpler prediction function.

**Nu**: since in R, the type could transfer to nu-classification as well, we could change this value.

For nu-classification, the dual formulation becomes

$$maxW(\alpha)=-\frac{1}{2}\sum_{i,j=1}^{n} \alpha_{i}\alpha_{j}y_{i}y_{j}k(x_{i},x_{i}))$$

$$s.t.0\leq\alpha_{i}\leq\frac{1}{n}(i=1,2,...,n)$$

$$\sum_{i=1}^{n} \alpha_{i}y_{i}=0$$

$$\sum_{i=1}^{n} \alpha_{i}\geq v.$$

The parameter $v$ has the interesting property of being an upper bound on the training error and a lower bound on the fraction of support vectors found in the data set. Thus, controlling the complexity of the classification function build by the SVM, see [13, 18, 41, 42] for details.

## Machine learning with Bayesian additive regression trees (BARTMACHINE)

BART is a Bayesian approach to nonparametric function estimation using regression trees. BART can be considered a sum-of-trees ensemble, with a novel estimation approach relying on a fully Bayesian probability model.

As a Bayesian model, BART consists of a set of priors for the structure and the leaf parameters and a likelihood for data in the terminal nodes. The aim of the priors is to provide regularization, preventing any single regression tree from dominating the total fit. BARTMACHINE introduces many new features for data analysis using BART.

$Model component$:

$$Y=f\left( x \right)+\epsilon\approx T_{1}^{M}\left( X \right)+T_{2}^{M}\left( X \right)+...+T_{m}^{M}\left( X \right)+\epsilon,\epsilon\sim N_{n}\left( 0,\sigma^{2}I_{n} \right),$$

where Y is the $n\times1$ vector of responses, X is the n×p matrix (the predictors column-joined) and $\epsilon$ is the $n\times1$ vector of noise. M is the number of the distinct regression trees, each composed of a tree structure, denoted by T, and the parameters at the terminal nodes, denoted by M. Hence $T^{M}$ represents an entire tree with both its structure and set of leaf parameters. The set of the tree's leaf parameters is denoted as $M_{t}=\mu_{t,1},\mu_{t,2},...,\mu_{t,b_{t}}$ where $b_{t}$ is the number of terminal nodes for a given tree. The observation's predicted value is the sum of the m leaf values arrived at by recursing down all m trees.

$Priors and likelihood$: The prior for the BART model has three components:

- The tree structure itself
- The leaf parameters given the tree structure
- The error variance $\sigma^{2}$ which is independent for the tree structure and leaf parameters

Assume that the conditional independence of the leaf parameters given the tree's structure.

$$P\left( T_{1}^{M},\ldots,T_{M}^{M},\sigma^{2} \right)=\left[ \prod^{t} P\left( T_{t}^{M} \right) \right]P\left( \sigma^{2} \right)=\left[ \prod^{t} P\left( M_{t} | T_{t} \right)P\left( T_{t} \right) \right]P\left( \sigma^{2} \right)=$$

$$=\left[ \prod^{t} \prod^{l} P\left( \mu_{(}t,l \right)\left| T_{t} \right)P\left( T_{t} \right) \right]P\left( \sigma^{2} \right).$$

$P(T_{t})$: This prior component affects the locations of nodes within the trees.

**Node depth** represents the distance from the root. Nodes at depth $d$are nonterminal with prior probability $\alpha(1+d)^{-\beta}$,where $\alpha\in(0,1)$,and $\beta\in[0,\infty]$. This component of the tree structure prior aims to enforce shallow tree structures, thereby limiting complexity of any single tree and resulting in more model regularization. The default number for this is $\alpha=0.95,\beta=2$.

For nonterminal nodes, unlike original formulation, where each available predictor has equal probability to be selected, in BART, this is relaxed to allow for a **generalized Bernoulli distribution**, where the user could specifies $p_{1},p_{2},...,p_{p},(\sum_{j=1}^{p} p_{j}=1)$, where each denotes the probability of the j^th^ variable being selected a priori.

$P(M_{t}|T_{t})$: This prior component controls the leaf parameters. When giving the set of terminal nodes, each terminal node has a leaf parameter representing the "best guess" of the response in this partition of predictor space. The prior on each of the leaf parameters is given as:

$\mu_{l}\sim N\left( \frac{\mu_{\mu}}{m},\sigma_{\mu}^{2} \right), \mu_{\mu}=(\frac{y_{min}+y_{max}}{2})$.

The variance $\sigma_{\mu}^{2}$ is chosen in order to make the range center plus or minus k variance cover 95% of the provided response values in the training set. The default value of k is 2. The aim of this prior is to provide model regularization by shrinking the leaf parameters toward the center of the distribution of the response. The larger the value of k, the smaller the value of $\sigma_{\mu}^{2}$, resulting in more model regularization.

$P(\sigma^{2})$: this component of prior is on the error variance and is chosen to be $\sigma^{2}\sim InvGamma(\frac{\nu}{2},\frac{\nu\lambda}{2})$.

The default values of $\alpha,\beta,k,\nu$ has good performance, but optimal tuning can be achieved via cross-validation. Along with a set of priors, BART specifies the likelihood of responses in the terminal nodes.

They are assumed a priori normal with the mean being the "best guess" in the leaf at the moment and variance being the best guess of the variance at the moment, $y_{l}\sim N(\mu_{l},\sigma^{2})$.

$Posterior distribution and prediction$: A key feature of BART is to employ a form of "Bayesian backfitting", where the jth tree is fit iteratively, holding all other m-1 trees constant by exposing only the residual response that remains unfitted:

$$R_{-j}=y-\sum^{t\neq j} T_{t}^{m}\left( X \right).$$

The procedure is shown as followed:

$$T_{1}|R_{-1},\sigma^{2}$$

$$M_{1}|T_{1},R_{-1},\sigma^{2}$$

$$T_{2}|R_{-2},\sigma^{2}$$

$$M_{2}|T_{2},R_{-2},\sigma^{2}$$

$$......$$

$$T_{m}|R_{-m},\sigma^{2}$$

$$M_{m}|T_{m},R_{-m},\sigma^{2}$$

$$\sigma^{2}|T_{1},M_{1},...T_{m},M_{m},\epsilon.$$

First, proposing a change to the first tree's structure T, which is accepted or rejected via a Metropolis-Hastings step, since the posterior of the tree structure does not depend on the leaf parameters. The revising includes small perturbations to the tree structure: growing a terminal node by adding two child nodes, pruning two child nodes, or changing a split rule.

Given the tree structure, the posterior then revise the leaf parameters. This procedure progresses iteratively for each tree, using the updated set of partial residuals $R_{-j}$. The posterior of each of the leaf parameters in M is conjugate normal with its mean being a weighted combination of the likelihood and prior parameters.

Finally, conditional on the updates set of tree structure and leaf parameters, a draw from the posterior of $\sigma^{2}$ is made based on the full model residuals

$$\epsilon=y-\sum_{t=1}^{m} T_{t}^{m}\left( X \right).$$

All these steps represent a single Gibbs iteration. Generally, no more than 1,000 iterations are needed as burn-in (the default value of burn-in node is 250). a single predicted value f(x) can be obtained by taking the average of the posterior values and a quantile estimate can be obtained by computing the appropriate quantile of the posterior values.

$Advantages$:

- BARTMACHINE is fully parallelized during the model creation, prediction, and many of the other features.
- The BARTMACHINE package also implements the variable selection procedures, which are best applied to data problems where the number of covariates influencing the response is small relative to the total number of covariate.

Like RandomForest algorithm, the main parameters we could use to expand this algorithm is num_trees: the number of trees to be grown in the sum-of-trees model (see [19] for details).

## SuperLearner

The super learner function takes a training set pair (X, Y) and returns the predicted values based on a validation set. With the V-fold cross validation theorem, this function could extent the candidate learner selector to include weighted averages of the candidate learners.

$Algorithm$:

Step 0: train each candidate learner on entire dataset.

Step 1: Split data into v blocks.

Step 2: train each candidate learner.

Step 3: predict the outcomes in the validation block based on the corresponding training block candidate learner.

Step 4: model selection and fitting for the regression of the observed outcome onto the predicted outcomes from the candidate learners.

$$E\left( Y | Z \right)=m\left( z;\beta\right),$$

where $\beta$ is the coefficient of each candidate learner and $z$ represents learners.

Step 5: evaluate super learner by combining predictions from each candidate learner with $m(z;\beta)$.

Usually, the default method on estimating the coefficients for the super learner and the model to combine the individual algorithms in the library is NNLS.

$NNLS$: The Lawson-Hanson algorithm for non-negative least squares solves the following problem:

$$argmin_{x}\parallel Ax-b\parallel_{2},x\geq0$$

$$A\in R^{m\times n},x\in R^{n},b\in R^{m}.$$

In fact, by using SuperLearner, we could predict m subjects by n methods and b is the real value of this m subjects. With NNLS, we could find optimal x (the coefficients of different algorithms), which lead to min error. In R, there always has a prescreen algorithms as well, which first rank the variables in X based on either a univariate regression p-value of the randomForest variable importance. A subset of the variables in X is selected based on a pre-defined cut-off. With this subset of the X variables, the algorithm in SL.Library are then fit in.

$Value$ - call: the configuration of this SuperLearner.

- libraryNames: a character vector with the names of the algorithms in the library, including the risk and coefficient for them.
- SL.library: returns in the same format as SL.librart in the argument. This will help us to have a clear mind about which algorithms are included in this SuperLearner function.
- SL.predict: the predict value from the super learner.
- coef: the coefficients for all the algorithms including in the SL.library. With the help of **coef**, we could compare the (weighted) parameters of each algorithm in the SL.library when obtaining the final super learner predicted value. Undoubtedly, the algorithm, whose parameter is larger than others, plays a more important role in predicting procedure, which means that this algorithm has fit better for this data set than others
- library.predict: a matrix with the predicted values from each algorithm in the SL.library.
- Z: a matrix with the cross-validation predicted values from each algorithm in the SL.library.
- cvRisk: a numeric vector with the v-fold cross-validated risk estimate for each algorithm in the SL.library without the the CV risk estimate for the SuperLearner.
- family: return the information about the family and link function. Through this, we could check different variables' family and, maybe, make further improvements by changing their link functions.
- futLibrary: a list with the fitted objects for each algorithm in SL.library on the full training data set.
- varNames: a character vector with the names of the variables in X.
- validRows: a list containing the row numbers for the v-fold cross-validation step.
- method: the method that used to combine algorithms in the SL.library to get SuperLearner prediction and compute error.We could change different methods and compare their computed error to get better prediction.
- whichScreen: a logical matrix indicating which variables passed each screening algorithm.
- Control: the control list.
- cvControl: the cvControl list.
- errorInCVLibrary: A logical vector indicating if any algorithms experienced an error within the CV step.
- errorsInLibrary: A logical vector indicating if any algorithms experienced an error on the full data (see [11] for details).

## Knockoff filter

Step 1: construct knockoffs

The knockoff filtering method doubles the number of original features by introducing a null-feature (${\overset{̃}{x}}_{j}$) corresponding to each original feature ($x_{j}$) in the design matrix X. These dummy (knockoff) variables serve as a "control group" that allows us to estimate the rate at which the regularized linear modeling generated false-positive results (i.e., a feature is declared statistically important, when in reality, it's not, just like the knockoffs represent null-data).

The knockoff variables are designed to

1. Preserve the correlation structure of the observed original (real) data, i.e.,${\overset{̃}{x_{j}}}^{T}\overset{̃}{x_{j}}=x_{j}^{T}x_{j}$, for all j,k.
2. Maintain the correlation between different original variables the same as those between knockoffs and distinct original features, i.e.,${\overset{̃}{x_{j}}}^{T}x_{k}=x_{j}^{T}x_{k}$, for $j\neq k$.

This the new (augmented) design matrix we use in the regularized linear model will be

$$[X\overset{̃}{X}]=[x_{1},x_{2},...,x_{n},\overset{̃}{x_{1}},\overset{̃}{x_{2}},...,\overset{̃}{x_{n}}]\in R^{k\times\left( 2n \right)}.$$

Let $\sum=X^{T}X$ represent the Gram matrix of the original features. According to the conditions above, we could ensure that $\overset{̃}{X_{T}}\overset{̃}{X}=\sum$ as well. Then we define $X_{T}\overset{̃}{X}=\sum-2\xi I_{n}$.

The dummy knockoff features are defined to satisfy the 2 conditions above and to enable the decomposition of the symmetrized augmented design matrix:

$$[X\overset{̃}{X}]^{T}[X\overset{̃}{X}]=\left( \begin{matrix} \sum& \left( \sum-2\xi I_{n} \right) \\ \left( \sum-2\xi I_{n} \right) & \sum\end{matrix} \right),$$

where the following definitions ensure the satisfaction of the two by-design conditions on the knockoffs above:$\sum=X^{T}X\sim\xi I_{n}$, is full rank, the knockoff features are derivatives of their real counterparts $\overset{̃}{X}=X(I_{n}-2\xi\sum)+UC$, the matrix $C^{T}C$ can be factorized using Cholesky decomposition into a lower triangular matrix (C) and its transpose ($C^{T}$), and U is an orthonormal matrix ($U^{T}U=I$) that is orthogonal to the original feature matrix $X(tr(UX^{T})=0)$.

Step 2: Calculate statistics for each pair of original and knockoff variables

We apply the LASSO model, and $l_{1}$ norm penalized regression to estimates the coefficients $\beta$.

$$\overset{̃}{\beta}(\lambda)=argmin_{b}\frac{1}{2}\parallel y-(X\overset{̃}{X})b)\parallel_{2}^{2}+\lambda\parallel b_{1}\parallel.$$

With this model, we could conclude that if variables $X_{j}$ enters the LASSO model early and it does so before its knockoff copy $\overset{̃}{X_{j}}$, then it means that this variable belongs to the model. Otherwise, it should not include to the model. Hence, we take $Z_{j}$ to be the point $\lambda$ on the lasso path at which feature $X_{j}$ first enters the model, $Z_{j}=sup_{\lambda}:\overset{̃}{\beta_{j}}(\lambda)$. $Z_{j}$ is small for those null variables.

We use the following test statistic is used to determine the significance of each of the n features in the design matrix X:

$$W_{j}=max\left( z_{j},\overset{̃}{z_{j}} \right)\cdot sgn\left( z_{j}-\overset{̃}{z_{j}} \right).$$

Step 3: Calculate a data-dependent threshold for the statistics.

To assess the performance of the knockoff filtering test, we use the false discovery rate (FDR):

$$\underset{false discovery rate}{\underset{⏟}{FDR}}=\underset{Expectation}{\underset{\underbrace{}}{E}}\left( \frac{number of false positives}{\underset{false discovery proportion}{\underset{\underbrace{}}{total number of selected features}}} \right).$$

Letting $q$ stands for the target FDR.

There has one theorem makes sure that this method could control a quantity nearly equal to the FDR.

**Theorem 1**. for any $q\in[0,1]$, the knockoff method satisfies:

$$E\left[ \frac{the number of j:\beta_{j}=0 and j\in\overset{̃}{S}}{the number of j:j\in\overset{̃}{S}+q^{-1}} \right]\leq q,$$

where the expectation is taken over the Gaussian noise z in the model, while treating X and $\overset{̃}{X}$ are fixed.

**Theorem 2**. for any $q\in[0,1]$, the knockoff+ method satisfies:

$$E\left[ \frac{the number of j:\beta_{j}=0 and j\in\overset{̃}{S}}{the number of j:j\in\overset{̃}{S}\vee1} \right]\leq q,$$

where the expectation is taken over the Gaussian noise z in the model, while treating X and $\overset{̃}{X}$~ are fixed.

Summarizing, the knockoff algorithm involves the following steps:

1. Augment the design matrix by introducing hat fake knockoff features.
2. Fit in the regularized linear model and obtain the estimates$[\beta_{1},\beta_{2},...,\beta_{n},\overset{̃}{\beta_{1}},\overset{̃}{\beta_{2}},...,\overset{̃}{\beta_{n}}]$via LASSO.
3. For each of the 2n features,$[x_{1},x_{2},...,x_{n},\overset{̃}{x_{1}},\overset{̃}{x_{2}},...,\overset{̃}{x_{n}}]$ , compute the statistical significance of their corresponding effect sizes (obtained in step 2) using parametric or non-parametric test for $H_{o}:\beta=0$ vs.$H_{\alpha}:\beta\neq0.$
4. Use the $[\overset{̃}{\beta_{1}},\overset{̃}{\beta_{2}},...,\overset{̃}{\beta_{n}}]$, as proxies of the knockoff features $[\overset{̃}{x_{1}},\overset{̃}{x_{2}},...,\overset{̃}{x_{n}}]$ to identify real vs. stochastic effects (the knockoff features serve as control variables (null effects), relative to the default $\alpha=0.05$.
5. Report the (real) features $[x_{(j_{1})},x_{(j_{2})},x_{(j_{3})},.,x_{(j_{m})}]$ that represent pairs of significant $x_{j}$ and insignificant features, according to the $W_{j}$ statistics.
6. This protocol guarantees that the expected false discovery rate for the reported features (step 5) would be within the predefined false positive rate $\alpha$=0.05 (see [6] for details).

## Confusion Matrix

A breakdown of predictions into a table showing correct predictions (the diagonal) and the types of incorrect predictions made (what classes incorrect predictions were assigned). The confusion matrix function (i.e., confusionMatrix()) calculates a cross-tabulation of observed and predicted classes with associated statistics, such as sensitivity, specificity, prevalence, PPV- positive predictive value =, NPV- negative predictive value, Detection Rate, Detection Prevalence, Balanced Accuracy.

The overall accuracy and unweighted Kappa statistic (or Cohen's kappa, a measure of classification accuracy normalized by the imbalance of the classes in the data) are calculated. A p-value from McNemar's test is also computed using the function McNemar.test (which can produce NA values with sparse tables). The overall accuracy rate is computed along with a 95 percent confidence interval for this rate (using the function binom.test) and a one-sided test to see if the accuracy is better than the "no information rate," which is taken to be the largest class percentage in the data. It also returns Precision (a measure of a classifiers exactness), Recall (a measure of a classifiers completeness) and the F1 Score (or F-score), which is a weighted average of precision and recall, see [43-46] for details.
