# Supplementary material for: Controlled feature selection and compressive big data analytics: Applications to biomedical and health studies
Source: PLoS One. 2018 Aug 30;13(8):e0202674. doi: 10.1371/journal.pone.0202674 (PMC6116997; doi:10.1371/journal.pone.0202674)
Supplement: S3 Text — Additional Results. (DOCX) [file pone.0202674.s003.DOCX]

Controlled Feature Selection and Compressive Big Data Analytics: Applications to Biomedical and Health Studies

Simeone Marino, Jiachen Xu, Yi Zhao, Nina Zhou, Yiwang Zhou, Ivo D. Dinov

**SUPPLEMENTARY INFORMATION**

# S3 Text: CBDA complete set of results

Due to the large number of experiments and the many different specifications, the complete set of results for the binomial and null datasets are shown at the following link:

[https://github.com/SOCR/CBDA](https://github.com/SOCR/CBDA%20)  (see releases/tag/v0.1-alpha)
