# Supplementary material for: Controlled feature selection and compressive big data analytics: Applications to biomedical and health studies
Source: PLoS One. 2018 Aug 30;13(8):e0202674. doi: 10.1371/journal.pone.0202674 (PMC6116997; doi:10.1371/journal.pone.0202674)
Supplement: S4 Text — (DOCX) [file pone.0202674.s004.DOCX]

Controlled Feature Selection and Compressive Big Data Analytics: Applications to Biomedical and Health Studies

Simeone Marino, Jiachen Xu, Yi Zhao, Nina Zhou, Yiwang Zhou, Ivo D. Dinov

**SUPPLEMENTARY INFORMATION**

# S4 Text: Mathematical Formulation of CBDA

### Step 1 Data Cleaning

Let’s label each original dataset as $DSorigin_{p}(p=1,2,...,n)$, and define a data cleaning algorithm as C. Then. $DSclean_{p}=C(DSorigin_{p}),p=1,2,...,n$.

### Step 2 Data Harmonization

Define a data harmonization algorithm by H. Then, $DS_{p}=H(DSclean_{p}),p=1,2,...,n$, where each $DS_{p}=[X_{p},Y_{p}],p=1,2,...,n$ has the same dimension.

### Step 3 Data Aggregation and Selection of Prediction Dataset

We define the large dataset $B=[X,Y]\in R^{N\times K}$, which is an aggregation of $DS_{p},p=1,2,...,n$,and where $N=n+m$ represents the total number of cases and $K$ represents the total number of features.

We then sample $m$ cases for validation, with $m<<n<N$. The large dataset $B$ is then partitioned in the following 4 datasets: $\left[ X_{temp},Y_{temp},X_{val},Y_{val} \right]$, where $\left[ X_{temp},Y_{temp} \right]$ is the data for training, with $X_{temp}\in R^{n\times K}$ , $Y_{temp}\in R^{n\times1}$ and $\left[ X_{val},Y_{val} \right]$ is the data for validation, with $X_{val}\in R^{m\times K}$ , $Y_{val}\in R^{m\times1}$.

Our goal is to find the dictionary matrix $\Phi$ to map the most important features for our predictive model.

### Step 4 Random Sampling

As described earlier in the Methods section, for each sample $j,(j=1,2,3,...,M)$, we sample cases and features according to our CSR (cases sampling range) and FSR (features sampling range). Thus, for each sample $j$ We have:

$\left[ X^{j},Y^{j},X_{val}^{j},Y_{val} \right]$,

where $X^{j}\in R^{n_{j}\times k_{j}},Y^{j}\in R^{n_{j}\times1},X_{val}^{j}\in R^{m\times k_{j}},Y_{val}\in R^{m\times1}$, with $k_{j}<<K$ and $n_{j}<<n<N$.

### Step 5 Data Imputation, Scaling and Re-balancing (optional)

This step is optional and may or may not be necessary depending on the specific case-study.

### Imputation

If $X^{j}$ has missing values as $X_{miss}^{j}$, we define R function 'missForest' [24] as MF, then we denote

$MF:R^{n_{j}\times k_{j}}\to R^{n_{j}\times k_{j}}$*,* and $X^{j}:=MF(X_{miss}^{j})$*,* here $X^{j}$ is a complete data sample*.*

### Scaling

If $X^{j}$ needs to be normalized as , we define a normalization function N. (Normalization functions are different, which to choose depends on demands). Here we take a normalization function for example, which makes each column average equal to 0 and the corresponding mean square equal to 1:

$N:R^{n_{j}\times k_{j}}\to R^{n_{j}\times k_{j}}$,,

then the m^th^ row and p^th^ column element in $X^{j}$ can be expressed as:

.

### Cohort Re-balancing

If the data sample is imbalanced as , we use a statistical rebalancing strategy, like the Synthetic Minority Over-sampling Technique (SMOTE) [26] to approximately equalize the group sizes. Let’s denote that by:

, where .

### Step 6 SuperLearner Algorithm

We define SuperLearner algorithm as ML:

$ML_{j}(C^{j}):R^{n_{j}\times k_{j}}\times R^{n_{j}\times1}\times R^{m\times k_{j}}\to R^{m\times1}$,

.

Here we separate the SuperLearner algorithm step into two parts (learning and validation) to obtain a clearer understanding or its effects.

### Learning step

Let's define the matrix $X\in$ **X** and the vector $Y\in$**Y**. Here **X**$=R^{n_{j}\times k_{j}}$ and **Y**$=R^{n_{j}\times1}$ (column vector).

We then define $A^{*}$ as the dual space of A, here $A=R^{1\times k_{j}}$,and $A^{*}$ includes a finite set of statistical learning algorithms as:$\forall F\in A^{*}$, $F:R^{1\times k_{j}}\to R$.

We then define the Descartes product between X and Y as: **X** $\bigotimes$**Y**. The element in **X** $\bigotimes$ **Y** has the form of (X, Y). We can express the black box learning process as a function $\phi$:

$\phi:$**X** $\bigotimes$ **Y** $\to A^{*}$, $\phi((X,Y))(a)=F(a)$, where $a\in A$.

For each $j$, $\phi((X^{j},Y^{j}))(a)=F^{j}(a)$. As an example, we can think of $\phi$ as the optimization algorithm embedded into our SuperLearner, with $A^{*}$ the set of algorithms specified in the SuperLearner library.

### Validation step

After we obtain the $F^{j}$, we apply it to $X_{val}^{j}$. Namely, . Here, $F^{j}$ is function on $X_{val}^{j}$ acting one column at a time, thus $F^{j}$ is a function on $R^{1\times k_{j}}$ outputting .

### Step 7 Ranking

We compare the with the $Y_{val}$ using a performance metric $\tau$ (e.g., accuracy, MSE, etc.). Let’s denote the performance by:

.

For example, when $\tau$ is MSE, $c_{j}=MSE_{j}$, and when $\tau$ is Accuracy, $c_{j}=Accuracy_{j}$. Then we apply the following operators:

1. Rank all the $\{c_{j}{\}}_{j=1,2,...,M}$.
2. Select of the top $\{c_{j}{\}}_{j=1,2,...,M}$.

These two steps may be denoted by the following operator: $\{c_{(j)}{\}}_{j=1,2,...,q}=O^{q}(\{c_{j}\})$.

### Step 8 Feature mining and Inference

Let’s define $\Omega$ to be the set of all the features $f_{i}$, then $\Omega=\{f_{i}\},i=1,2,...,K$. The next step involves first counting and then ranking the occurrences of each $f_{i}$ across the top $\{c_{(j)}{\}}_{j=1,2,...,q}$. This is expressed by:

1. Setting 1 if the feature occurs and 0 if not, here $j=1,2,...,q$.

$$s_{j}^{(i)}=Dirichlet_{j}\left( f_{i} \right)=\left\{ \begin{matrix} 0, f_{i}\notin sample j \\ 1, f_{i}\in sample j \end{matrix} \right..$$

2. Counting occurrences of each $f_{i}$ across the top q$\{c_{(j)}{\}}_{j=1,2,...,q}$ by:

$S_{i}=\underset{j}{Sum}(s_{j}^{(i)}),i=1,2,...,K$.

1. Ranking the occurrences of each $f_{i}$ and obtain new ranked feature set:

$S_{(i)}=O^{K}(S_{i}),i=1,2,...,K$.

$\Omega^{*}=\{f_{1},...,f_{i},...,f_{K}\}$.

We can also formalizing the above protocol as follows: Set up a matric as $F\in R^{q\times K}$, where the rows represent the top samples and the columns represent the features. Then, $\forall b_{ji}\in F,b_{ji}=s_{j}^{(i)}$ and $S_{i}=\sum_{j}^{q} b_{ji},i=1,2,...,K$.

The inference step takes as input the $\Omega^{*}$ defined above and our goal in the inference step is to define the optimal dimension of the dictionary matrix $\Phi$, where we plug the most important features into our predictive model. One way to automate the selection of how many features to include in our predictive model is to find the minimal set of features $l$ that ensures the best performance metric $\tau$. To do so, we run the following post-optimization feature mining scheme on $\Omega^{*}$ the top features. We can make this step more efficient by considering an example of choosing 5 features at a time within the features index $p$:

- - for $p=1$ we select the top 5 features $k_{1}^{*}=5$
  - for $p=2$ we select the top 10 features $k_{2}^{*}=10$
  - ....., and so on.
  - for we select the top $P$ features (stop here).

Here $k_{p}^{*}$ represents the number of top features that we chose and $p=\frac{P}{5}$. Alternatively, we can simply add one feature at a time, starting from a minimal set of features that we want to be built into our predictive model (e.g., the minimal model will start from the top 5 features from $\Omega^{*}$). Also, $P$ can be chosen to be a function of the total number of features $K$ (e.g., $5+log(K)$, with $5$ being the number of features in our minimal predictive model).

Then we have:

$Y_{val}^{p}\in R^{m}.$

If the performance metric is given by $\tau(Y_{val}^{p},Y_{val})$, we can select the $Best_{p=1,2,.....,l}(\tau(Y_{Val}^{p},Y_{val}))$. For instance, if we use $\tau=Accuracy=A$ as our performance metric, we have:

,

and we can select the set of features to include in the “best” predictive model by following the stopping criteria outlined below. We can also plot $A(Y_{Val}^{p},Y_{val})$ against the number of features $k_{p}^{*}$ and use the plot to guide our selection of the “best” set of features.

A similar strategy can be implemented if we use $\tau=MSE$ as our performance metric, where we have:

$MSE(Y_{val}^{p},Y_{val})=\frac{1}{m}\parallel Y_{val}^{p}-Y_{val}\parallel_{2}^{2}$.

### *Stopping Criteria*

To avoid overfitting, we can implement several alternative strategies. For example, if we use $\tau=Accuracy=A$ as our performance metric, we can set a minimal threshold for $A$ (i.e., $A>70$) and

1. Stop if $A(t+1)<=A(t)$, where $A(t+1)$ has 1 more features than $A(t)$ in the predictive model
2. Stop if $A(t+1)/A(t)-1<\lambda$ (with $\lambda=$ 0.1, 0.05 or 0.01). This step ensures at least a 10% or 5% or 1% improvement.

If we use $\tau=MSE$ as our performance metric, we can implement the following stopping criteria:

1. Stop if $MSE(t+1)\geq MSE(t)$, where $MSE(t+1)$ has 1 more features than $MSE(t)$ in the predictive model.
2. Stop if $MSE(t+1)/MSE(t)>F(n_{1},n_{2},\alpha)$, where $F$ represents the F of Fisher with degrees of freedom $n_{1}$ and $n_{2}$, respectively, and $\alpha$ the level of significance of the test. This test ensures that the 2 $MSEs$ are significantly different from each other. In other words, we want to be sure that adding an extra feature to our predictive model will make a significant impact in reducing the metric $\tau=MS$.
