# Supplementary material for: Controlled feature selection and compressive big data analytics: Applications to biomedical and health studies
Source: PLoS One. 2018 Aug 30;13(8):e0202674. doi: 10.1371/journal.pone.0202674 (PMC6116997; doi:10.1371/journal.pone.0202674)
Supplement: S1 Table — (DOCX) [file pone.0202674.s005.docx]

Controlled Feature Selection and Compressive Big Data Analytics: Applications to Biomedical and Health Studies

Simeone Marino, Jiachen Xu, Yi Zhao, Nina Zhou, Yiwang Zhou, Ivo D. Dinov

**Table S1: CBDA Robustness.** **Table S1.A** depicts the level of importance of the features in each ranking based on Accuracy and MSE. Among them, the true features selected by Accuracy are marked as light green, and the true features selected by MSE are marked as dark green, respectively. For each experiment (i.e., Rep1,…, Rep10), higher proportions of (light or dark) green cells in the corresponding column indicate higher reliability in detecting the signal in the real features in the data. The order by which the features are listed in **Table S1** reflects the level of importance of the features in each ranking based on Accuracy and MSE. For example, for replication 4 (Rep4 columns), the top 3 features selected are 300, 800 and 400 if we use the Accuracy metric, and 800, 300 and 900 if we use the MSE metric. We highlight in green the "true" features selected within the top 10. These results confirm that the MSE metric is the more appropriate for feature mining in the CBDA protocol (more coverage of "true" features). **Table S1.B** is similar to this one but it displays the 10 replications on the Binomial dataset with 300 cases and 100 features (true features: 10, 20, 30, 40, 50, 60, 70, 80, 90 and 100).

**Table S1.A: Assessing CBDA robustness using the Binomial dataset 3 (n=300, p=900):**

based on true features: 1, 100, 200, 300, 400, 500, 600, 700, 800, 900.

| **Rep1** | | | **Rep2** | | | | **Rep3** | | | | **Rep4** | | | | **Rep5** | | |
| --- | --- | --- | --- | --- | --- | --- | --- | --- | --- | --- | --- | --- | --- | --- | --- | --- | --- |
| ***Acc*** | ***MSE*** | | ***Acc*** | | ***MSE*** | | ***Acc*** | | ***MSE*** | | ***Acc*** | | ***MSE*** | | ***Acc*** | | ***MSE*** |
| **300** | **300** | | **300** | | **300** | | **300** | | **800** | | **300** | | **800** | | **300** | | **800** |
| **800** | **800** | | **800** | | **800** | | **800** | | **300** | | **800** | | **300** | | **800** | | **300** |
| **400** | **400** | | **400** | | **400** | | **400** | | **900** | | **400** | | **900** | | **400** | | **400** |
| **900** | **900** | | **100** | | **100** | | **900** | | **100** | | **900** | | **100** | | **650** | | **900** |
| **650** | **1** | | **600** | | **600** | | **100** | | **400** | | **100** | | **400** | | **845** | | **500** |
| **100** | **100** | | **900** | | **900** | | **496** | | **500** | | **600** | | **500** | | **900** | | **100** |
| **700** | **700** | | **496** | | **500** | | **747** | | **200** | | **500** | | **600** | | **496** | | **600** |
| **496** | **600** | | **845** | | **700** | | **738** | | **1** | | **650** | | **1** | | **527** | | **700** |
| **500** | **500** | | **79** | | **1** | | **500** | | **600** | | **496** | | **700** | | **110** | | **1** |
| **1** | **845** | | **623** | | **200** | | **650** | | **623** | | **486** | | **200** | | **700** | | **496** |
| **Rep6** | | **Rep7** | | | | **Rep8** | | | | **Rep9** | | | | **Rep10** | | | |
| ***Acc*** | ***MSE*** | ***Acc*** | | ***MSE*** | | ***Acc*** | | ***MSE*** | | ***Acc*** | | ***MSE*** | | ***Acc*** | | ***MSE*** | |
| **300** | **300** | **300** | | **800** | | **300** | | **800** | | **300** | | **300** | | **300** | | **300** | |
| **800** | **800** | **800** | | **300** | | **400** | | **300** | | **800** | | **900** | | **800** | | **800** | |
| **100** | **100** | **900** | | **900** | | **800** | | **900** | | **900** | | **800** | | **400** | | **900** | |
| **400** | **900** | **400** | | **600** | | **900** | | **400** | | **400** | | **400** | | **900** | | **400** | |
| **900** | **400** | **100** | | **400** | | **100** | | **100** | | **100** | | **100** | | **100** | | **600** | |
| **650** | **600** | **600** | | **100** | | **496** | | **500** | | **700** | | **600** | | **600** | | **100** | |
| **1** | **1** | **706** | | **500** | | **878** | | **1** | | **650** | | **1** | | **529** | | **1** | |
| **409** | **500** | **650** | | **1** | | **409** | | **600** | | **765** | | **500** | | **486** | | **500** | |
| **556** | **200** | **138** | | **200** | | **500** | | **222** | | **600** | | **650** | | **764** | | **529** | |
| **700** | **700** | **764** | | **700** | | **222** | | **138** | | **222** | | **700** | | **805** | | **700** | |

**Table S1.B: Assessing CBDA robustness using the Binomial dataset (n=300, p=100):**

using true features: 10, 20, 30, 40, 50, 60, 70, 80, 90, 100.

| **Rep1** | | **Rep2** | | **Rep3** | | **Rep4** | | **Rep5** | |
| --- | --- | --- | --- | --- | --- | --- | --- | --- | --- |
| ***Acc*** | ***MSE*** | ***Acc*** | ***MSE*** | ***Acc*** | ***MSE*** | ***Acc*** | ***MSE*** | ***Acc*** | ***MSE*** |
| **80** | **70** | **32** | **70** | **70** | **70** | **20** | **70** | **80** | **70** |
| **70** | **80** | **70** | **32** | **32** | **60** | **80** | **80** | **90** | **80** |
| **90** | **90** | **60** | **30** | **60** | **30** | **90** | **100** | **20** | **100** |
| **55** | **100** | **30** | **90** | **80** | **32** | **10** | **10** | **70** | **10** |
| **32** | **32** | **90** | **100** | **10** | **90** | **32** | **90** | **60** | **90** |
| **30** | **60** | **80** | **60** | **30** | **10** | **60** | **60** | **32** | **50** |
| **76** | **50** | **57** | **10** | **90** | **80** | **22** | **20** | **62** | **60** |
| **10** | **10** | **21** | **80** | **57** | **100** | **55** | **32** | **10** | **20** |
| **20** | **55** | **100** | **20** | **20** | **50** | **83** | **55** | **15** | **32** |
| **60** | **20** | **20** | **57** | **41** | **57** | **40** | **83** | **79** | **30** |
| **Rep6** | | **Rep7** | | **Rep8** | | **Rep9** | | **Rep10** | |
| ***Acc*** | ***MSE*** | ***Acc*** | ***MSE*** | ***Acc*** | ***MSE*** | ***Acc*** | ***MSE*** | ***Acc*** | ***MSE*** |
| **70** | **70** | **32** | **70** | **32** | **70** | **60** | **70** | **60** | **70** |
| **60** | **60** | **60** | **90** | **60** | **30** | **80** | **80** | **32** | **30** |
| **80** | **100** | **20** | **32** | **20** | **90** | **55** | **60** | **70** | **100** |
| **30** | **80** | **57** | **80** | **70** | **32** | **20** | **100** | **30** | **32** |
| **90** | **90** | **90** | **60** | **30** | **60** | **10** | **10** | **80** | **60** |
| **100** | **30** | **80** | **10** | **57** | **100** | **67** | **90** | **80** | **10** |
| **32** | **10** | **40** | **30** | **90** | **20** | **20** | **32** | **90** | **90** |
| **10** | **32** | **70** | **100** | **15** | **80** | **32** | **20** | **57** | **57** |
| **20** | **50** | **34** | **20** | **80** | **10** | **100** | **55** | **100** | **40** |
| **40** | **40** | **30** | **57** | **60** | **57** | **79** | **20** | **10** | **50** |
