# Supplementary material for: Controlled feature selection and compressive big data analytics: Applications to biomedical and health studies
Source: PLoS One. 2018 Aug 30;13(8):e0202674. doi: 10.1371/journal.pone.0202674 (PMC6116997; doi:10.1371/journal.pone.0202674)
Supplement: S2 Table — (DOCX) [file pone.0202674.s006.docx]

Controlled Feature Selection and Compressive Big Data Analytics: Applications to Biomedical and Health Studies

Simeone Marino, Jiachen Xu, Yi Zhao, Nina Zhou, Yiwang Zhou, Ivo D. Dinov

## Table S2: Summary of ADNI data features

| **Variable Name** | **Variable Name** |  | **Legend** | | | | | | |  |  |
| --- | --- | --- | --- | --- | --- | --- | --- | --- | --- | --- | --- |
| Index | L_angular_gyrus | | |  |  | Clinical data | | | | |  |
| Case | R_angular_gyrus | | |  |  | Imaging data (56 ROI). See [Refs] for descriptions | | | | |  |
| Data | L_precuneus | | |  |  | Response/Outcome | | | | |  |
| siteKey | R_precuneus | | |  |  | Deleted features [after data wrangling] | | | | |  |
| \| subjectIdentifier \| L_superior_occipital_gyrus \| \| --- \| --- \| \| researchGroup \| R_superior_occipital_gyrus \| \| subjectSex \| L_middle_occipital_gyrus \| \| subjectInfo..item..DX.Group \| R_middle_occipital_gyrus \| \| subjectInfo..item..APOE.A1 \| L_inferior_occipital_gyrus \| \| subjectInfo..item..APOE.A2 \| R_inferior_occipital_gyrus \| \| visitIdentifier \| L_cuneus \| \| MMSCORE \| R_cuneus \| \| GDTOTAL \| L_superior_temporal_gyrus \| \| CDGLOBAL \| R_superior_temporal_gyrus \| \| NPISCORE \| L_middle_temporal_gyrus \| \| FAQTOTAL \| R_middle_temporal_gyrus \| \| studyIdentifier \| L_inferior_temporal_gyrus \| \| subjectAge \| R_inferior_temporal_gyrus \| \| weightKg \| L_parahippocampal_gyrus \| \| postMortem \| R_parahippocampal_gyrus \| \| seriesIdentifier \| L_lingual_gyrus \| \| Background \| R_lingual_gyrus \| \| L_superior_frontal_gyrus \| L_fusiform_gyrus \| \| R_superior_frontal_gyrus \| R_fusiform_gyrus \| \| L_middle_frontal_gyrus \| L_insular_cortex \| \| R_middle_frontal_gyrus \| R_insular_cortex \| \| L_inferior_frontal_gyrus \| L_cingulate_gyrus \| \| R_inferior_frontal_gyrus \| R_cingulate_gyrus \| \| L_precentral_gyrus \| L_caudate \| \| R_precentral_gyrus \| R_caudate \| \| L_middle_orbitofrontal_gyrus \| L_putamen \| \| R_middle_orbitofrontal_gyrus \| R_putamen \| \| L_lateral_orbitofrontal_gyrus \| L_hippocampus \| \| R_lateral_orbitofrontal_gyrus \| R_hippocampus \| \| L_gyrus_rectus \| cerebellum \| \| R_gyrus_rectus \| brainstem \| \| L_postcentral_gyrus \| \| R_postcentral_gyrus \| \| L_superior_parietal_gyrus \| \| R_superior_parietal_gyrus \| \| L_supramarginal_gyrus \| \| R_supramarginal_gyrus \| | | | |  | | |  |  |  | | |
