# Supplementary material for: Controlled feature selection and compressive big data analytics: Applications to biomedical and health studies
Source: PLoS One. 2018 Aug 30;13(8):e0202674. doi: 10.1371/journal.pone.0202674 (PMC6116997; doi:10.1371/journal.pone.0202674)
Supplement: S3 Table — (DOCX) [file pone.0202674.s007.docx]

Controlled Feature Selection and Compressive Big Data Analytics: Applications to Biomedical and Health Studies

Simeone Marino, Jiachen Xu, Yi Zhao, Nina Zhou, Yiwang Zhou, Ivo D. Dinov

**Table S3**: CBDA binomial classification results on the ADNI dataset. Performance assessment using confusion matrix and evaluation statistics.

|  | | **Reference** | |
| --- | --- | --- | --- |
| **Prediction** | | *AD* | *Normal* |
| *AD* | | 232 | 6 |
| *Normal* | | 18 | 244 |
| **Overall Statistics** | | | |
| *Accuracy* | | 0.952 [ 95% CI = (0.9294, 0.969)] | |
| *No Information Rate* | | 0.5 | |
| *P-Value* [Acc > NIR] | | <2e-16 | |
| *Kappa* | | 0.904 | |
| *McNemar's Test P-Value* | | 0.02474 | |
| **Sensitivity** | 0.828 | | |
| **Specificity** | 0.976 | | |
| **Positive Pred Value** | 0.9748 | | |
| **Negative Pred Value** | 0.9313 | | |
| **Precision** | 0.9748 | | |
| **Recall** | 0.928 | | |
| **F1** | 0.9508 | | |
| **Prevalence** | 0.5 | | |
| **Detection Rate** | 0.464 | | |
| **Detection Prevalence** | 0.476 | | |
| **Balanced Accuracy** | 0.952 | | |
